# Pooled image-base screening of mitochondria with microraft isolation distinguishes pathogenic mitofusin 2 mutations

**DOI:** 10.1038/s42003-022-04089-y

**Published:** 2022-10-25

**Authors:** Alex L. Yenkin, John C. Bramley, Colin L. Kremitzki, Jason E. Waligorski, Mariel J. Liebeskind, Xinyuan E. Xu, Vinay D. Chandrasekaran, Maria A. Vakaki, Graham W. Bachman, Robi D. Mitra, Jeffrey D. Milbrandt, William J. Buchser

**Affiliations:** 1 Department of Genetics, Washington University School of Medicine, St Louis, MO USA; 2 Functional Imaging for Variant Elucidation at the McDonnell Genome Institute, St Louis, MO USA

**Keywords:** Functional genomics, Cellular imaging, Mitochondria, Mutagenesis

## Abstract

Most human genetic variation is classified as variants of uncertain significance. While advances in genome editing have allowed innovation in pooled screening platforms, many screens deal with relatively simple readouts (viability, fluorescence) and cannot identify the complex cellular phenotypes that underlie most human diseases. In this paper, we present a generalizable *functional genomics* platform that combines high-content imaging, machine learning, and microraft isolation in a method termed “Raft-Seq”. We highlight the efficacy of our platform by showing its ability to distinguish pathogenic point mutations of the mitochondrial regulator Mitofusin 2, even when the cellular phenotype is subtle. We also show that our platform achieves its efficacy using multiple cellular features, which can be configured on-the-fly. Raft-Seq enables a way to perform pooled screening on sets of mutations in biologically relevant cells, with the ability to physically capture any cell with a perturbed phenotype and expand it clonally, directly from the primary screen.

## Abstract (web summary)

Raft-Seq is a generalizable pooled screening platform that combines high-content imaging, machine learning and microraft isolation, and enables efficient screening of genetic perturbations based on their impact on phenotypes.

## Introduction

The explosion of functional genomics in the past decade^1^ has enabled a massive shift in the study of the underlying genetics of human pathology. Even so, it is difficult to connect specific genetic mutations to disrupted cellular phenotypes, necessitating a detailed phenotyping-based *functional genomics* platform that can both screen large numbers of genetic perturbations/variants—so-called multiplexed assays of variant effects^2^—and work alongside methods of generating variant libraries, such as deep mutational scanning^3^.

To assess the impact of these variants on complex phenotypes, high-content imaging/screening^4–8^ is performed in an arrayed format. While some of these methods are particularly advanced^9–14^, methods under this framework are difficult to scale and infeasible for assaying combinatorial effects. Pooled genetic perturbation screens, on the other hand, have generally relied on enrichment analysis^9^ and cannot provide a one-to-one correspondence between a single cell and its phenotype. Beyond enrichment, simple phenotypes measurable by flow cytometry^10–12^, or phenotypes measurable by sequencing^13–15^ are possible. There are now platforms that use an imaging-based approach in pooled genetic perturbation screens and have been demonstrated on relatively simple phenotypes^16–18^ or on precise phenotypes that were known in advance^19,20^.

Although some platforms do isolate individual cells^21^, most genetic perturbation screens use a pooled population-level measurement as their endpoint^22^. The main challenge for such screens that operate on the single-cell level is mapping the perturbation back to each cell post-sequencing. For screens where the phenotype is measurable through sequencing, the perturbagen can be found simultaneously with the phenotype with single-cell resolution^13,15^, and newer methods have incorporated more advanced sequencing techniques to find additional data, such as surface protein presence^15^.

Pooled screening platforms that incorporate complex phenotypes only accessible through microscopy have required more involved approaches. Mapping phenotype to perturbation requires retaining information about the cell’s physical position, and there are a few strategies for accomplishing that. Some platforms use in situ sequencing (ISS)^23–25^ to generate sequencing results that contain positional data of a specific cell^19,26^ using specialized non-commercial sequencing rigs. Other platforms photoactivate endogenous fluorophores in targeted cells which are then put through FACS-seq^18,20,27^.

In this paper, we present Raft-Seq, a pooled screening platform that predicts individual cell perturbations from high-content imaging and machine learning. Raft-Seq improves on other platforms in several important ways: 1, it uses a microraft plate^28–30^ for context-aware isolation of identified cells 2, it can use vital dyes or stains, so no genetic modification of the cell is required other than the perturbation itself; 3, it is largely phenotype-agnostic, needing only knowledge of the broad physiology beforehand for stain selection and initial feature filtering; 4, it uses machine learning to identify perturbed cells, allowing the identification of complex cell-autonomous phenotypes; 5, It is able to connect genetic variation to disrupted cellular phenotypes in a 1:1 fashion; 6, it selects cells with high-viability and clonability, directly from the primary screen. The use of microraft plates for pooled screening has been demonstrated before^30^, and this manuscript builds on its use to make an extensible single-cell automated screening platform.

Here, we use the Raft-Seq platform to examine neurologically relevant mutations in the *MFN2* gene, which normally protects against cellular stress from damaged mitochondria by regulating mitochondrial fission and fusion^31^. Clinical *MFN2* mutations (pathogenic variants) primarily result in Charcot-Marie-Tooth Disease, the most common inherited neuromuscular disorder characterized by peripheral neuropathy with impairment of the central nervous system^32–36^. We find that the phenotype caused by pathogenic *MFN2* variants is distinct, but the difference is not adequately described by a single measurement/feature, necessitating a more complex feature analysis. Following the findings from the single perturbation experiments, we targeted a screening gRNA library across the *MFN2*-coding region to identify anomalous phenotypes caused by these mutations.

## Results

### Raft-seq is a microraft-based perturbation platform for screening complex phenotypes

**Fig. 1:**
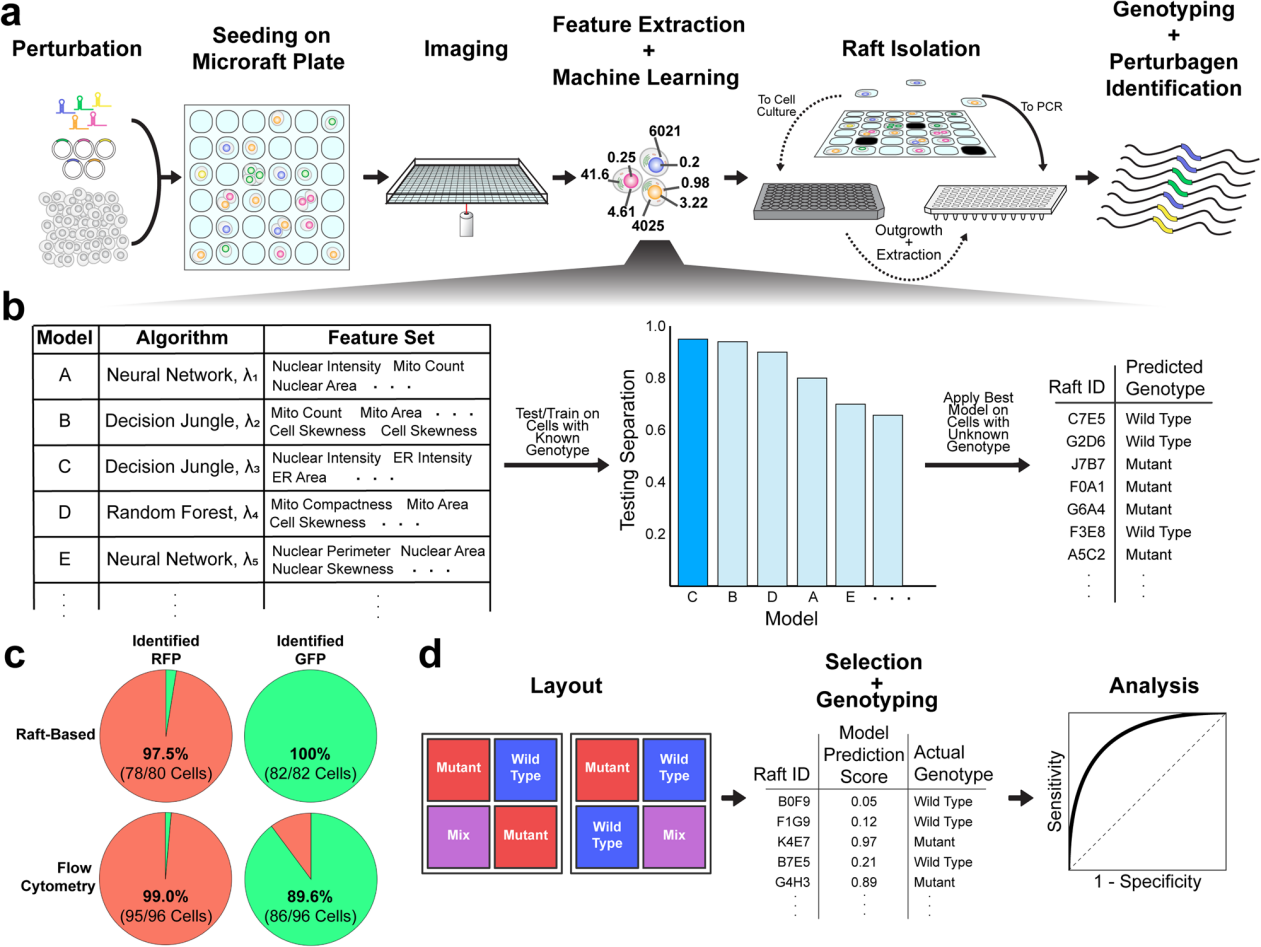
Raft-Seq experimental workflow. **a** A perturbagen library, primarily a lentiviral gRNA or plasmid overexpression library, is introduced to cells, which are then seeded onto a microraft plate. The plate is imaged, and cell feature data are then extracted from the resulting images and used to build machine-learning models. The trained model then selects rafts to either be isolated into a PCR plate for immediate analysis or a tissue culture plate for clonal expansion. Following isolation, the cells are genotyped. **b** Modeling workflow. Each model comprises a feature set and machine learning algorithm with different hyperparameters, represented here by λ. Each model is evaluated with training and testing cell data, and the model that shows a large separation between the ranked prediction curves for the test set and is deemed to have minimal overfitting is used to generate a list of cells (rafts) to pick, along with their predicted genotype. **c** Pie charts showing the composition of cells identified as either expressing GFP or RFP, after being separated by either the raft-based approach or flow cytometry. The color represents the expected appearance of the cells based on genotype (green = contains RFP gRNA, red = contains GFP gRNA), and the fractions/percentages are the amount in each group that is correctly identified. **d** Performance evaluation workflow. The blue and red wells represent the labeled cell populations and the purple wells represent the unlabeled population that is a mixture between the two. A model is used to select rafts that are then isolated and single-cell genotyped. The comparison between the prediction scores and the true genotype class generates a receiver operator characteristic curve to evaluate model efficacy.

The workflow of Raft-Seq can be summarized in five steps: perturbation, imaging, model building, isolation, and sequencing (Fig. 1a, b). In the first step, we introduce a set of genetic perturbations to a population of cells. The cells are then seeded onto a microraft plate, stained, and imaged on a high-throughput confocal microscope. We designed Raft-seq primarily using live-cell staining so that we could isolate viable cells. After imaging, the fluorescence data is used to compute cell/nuclear/organelle features with Cytiva’s INCarta software and are then compiled with metadata into a dataset. The resulting dataset is quality-checked (Methods: *Image Analysis and Quality Control*) and normalized prior to further data preprocessing and modeling. Several supervised models are trained, varying by algorithm, hyperparameters, and features (list in Supplementary Note 1), and the models are evaluated on several metrics using training and testing subsets of cells with known perturbation/genotypes *(*Methods: *Machine Learning and Model Generation / Modeling* Considerations). The best model determines the cells to be isolated.

Isolation is performed using the CellRaft Air System, where 100 × 100 µm microrafts containing cells of interest are automatically transferred to a 96-well plate, either for immediate genotyping or for initial outgrowth. Due to the time it takes to isolate each microraft, Raft-seq has a lower average throughput than other screening methods. In contrast to methods that use ISS^19,23^, Raft-seq physically isolates the cells which can be sequenced using commercial technology. Raft-seq associates a sequence with the specific image of cell(s), something not possible in methods that sort using FACS-Seq^18,20,27^. Previous screening experiments using microrafts^30^ isolated fixed cells, so Raft-Seq’s ability to capture individual live cells for growth alongside their original microscopy data is unique among pooled screening platforms.

As a preliminary test of raft-based imaging and isolation, we performed Raft-Seq on cells with dual genome-encoded RFP and GFP that had been given a gRNA to knock out either RFP or GFP. These cells were plated on a microraft plate as a mixture and were imaged. Using fluorescent marker intensity, we predicted the guide presence, isolated those cells, performed single-cell DNA sequencing to look for the edited genomic targets, and determined the true genotype. The correspondence between the predicted guide and the true guide was near perfect (Accuracy = 98.8%, *n* = 162) and comparable to flow-based single-cell sequencing run in parallel (Fig. 1c).

### Identifying subtle mitochondrial phenotypes in a mixed-variant pool

To validate the efficacy of Raft-Seq in a more complex screen, we attempted to separate a mixture of wild-type and mutant cells by reproducibly predicting a given cell’s *mutant status* determined by a set of features extracted from imaging data (with no fluorescent reporters, Fig. 1d). For this experiment, we set out to separate cells containing pathogenic *MFN2* mutations from those containing the wild-type *MFN2* cDNA.

We generated U2OS cell lines that each contain *MFN2* cDNA with single point mutations through lentiviral infection. Six clinically pathogenic *MFN2* variants^37^ and five control variants annotated as “benign” from ClinVar^38^ (Supplementary Fig. 1a).

To investigate the phenotypes caused by the *MFN2* variants, we assessed mitochondrial morphology and membrane potential using MitoTracker and TMRM (a tetramethylrhodamine analog), respectively^39^. FCCP, an oxidative phosphorylation uncoupler, was used to confirm that Mitotracker and TMRM were dosed at appropriate concentrations and were reporting on mitochondrial fluorescence^40^ (Supplementary Fig. 2).

A visual comparison of the *MFN2* cell lines demonstrates subtle phenotypic differences among them (Supplementary Fig. 1), specifically, perinuclear aggregation of mitochondria in the cells containing the pathogenic mutations. Mitochondrial aggregation and disrupted mitochondrial membrane potential are present in the mutants (Supplementary Figs. 1c, 3, 4). Although there are significant differences in the population of mutants, the features have largely overlapped distributions across genotypes. PCA and UMAP dimensionality reduction were performed on the full set of cellular image-based features (Supplementary Figs. 1d, 5) and each shows that using data from multiple features more reliably distinguishes cells with pathogenic mutations.

The goal was then to identify any of four pathogenic mutants (L76P, R94Q, P251A, R280H) from WT in an admixture of those cells. In the different wells of the microraft plate, we plated either 1) *MFN2* WT cells, 2) a mixture of the 4 *MFN2* mutants (in equal proportion), 3) a mixture of the wild-type and the 4 pathogenic cells at a wild-type:pathogenic ratio of 90:10, and 4) a similar mixture at a ratio of 50:50. We distinguish between the first two groups and the last two by calling the former (1,2) “labeled” and the latter (3,4) “unlabeled” to clarify for which cell populations the genotype class was known prior to imaging and analysis. Following the application of a set of stains (Methods: Staining), the cells were imaged, and their features were computed. Next, we designed classification models that best distinguished the labeled populations (*MFN2* WT vs. *MFN2* mutants).

Using the selected model, we chose cells for isolation from the unlabeled wells with additional control cells picked from the labeled wells (from a set of 30,682 cells that were imaged in 1.4 h). Over 1000 cells were then automatically and individually isolated into wells of several 96-well PCR plates (384 labeled control cells, 758 unlabeled cells). From there, the cells underwent amplicon library construction, multiplexing, and NGS, from which their genotypes were ascertained.

**Fig. 2:**
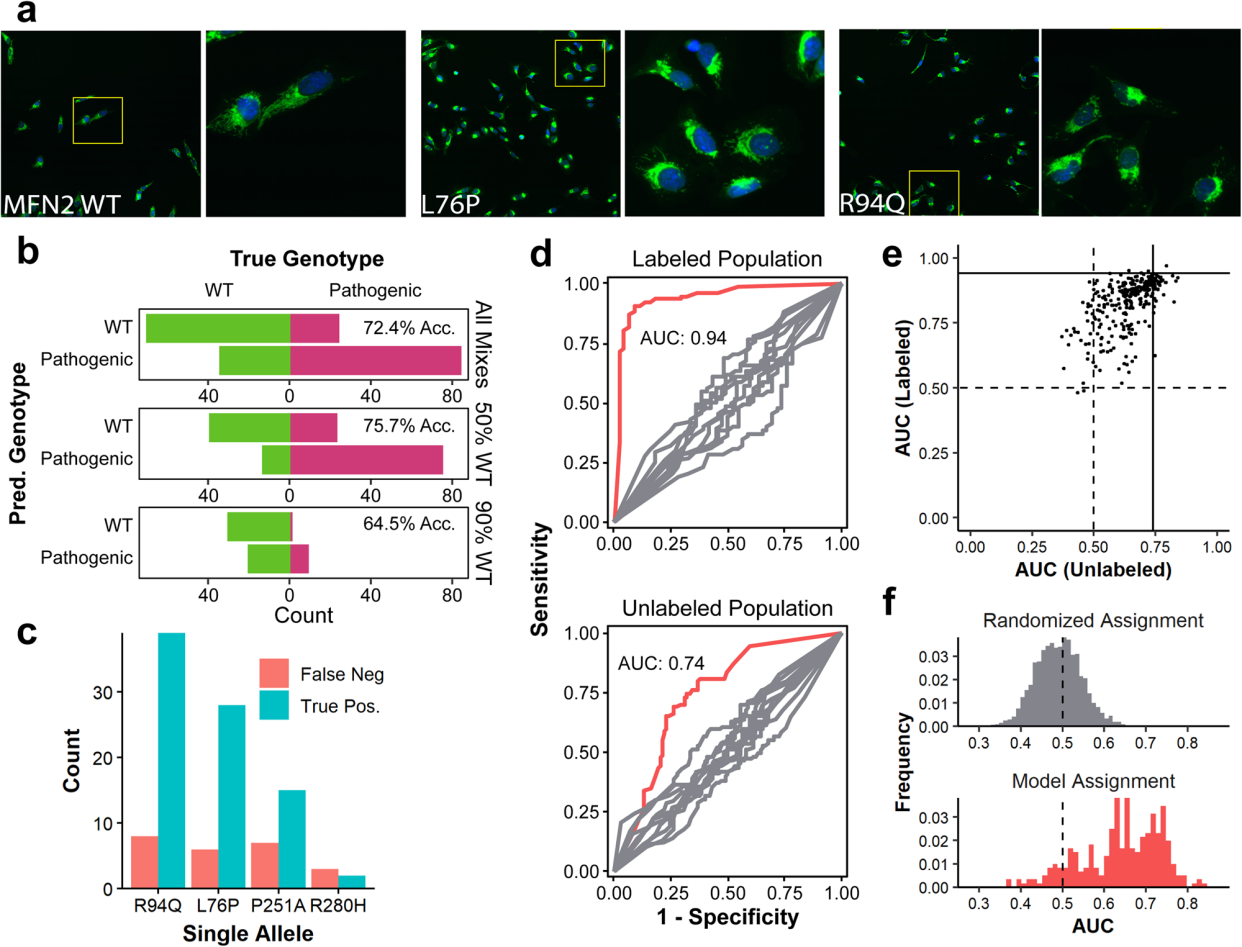
Raft-seq proof-of-principle results with *MFN2* pathogenic mutations. **a** Confocal images of mutant cells at two magnifications (blue = nuclear staining by Hoechst, green = mitochondria staining by MitoTracker). Subtle mitochondrial differences can be observed amongst the mutant populations. Known pathogenic mutants display perinuclear aggregation of mitochondria and a lack of mitochondrial spreading. In comparison, wild-type cells show ample mitochondrial spreading. Insets are 550 µm in width and height. **b** Confusion bar charts of predicted genotype vs. true genotype for the model used from cells in the WT/Pathogenic mixture. “All Mixes” refers to all cells isolated from the mixture, while “50% WT” and “90% WT” refer to a breakdown of the total according to whether the cells were isolated from the well containing 50% or 90% wild-type cells, respectively. **c** A bar chart of picked mutants separated by allele and counting the final status of individual cells. **d** ROC Curves generated using the best model identified *a posteriori* separated by data generated from cells picked from control pure wells (Labeled) and from mix wells (Unlabeled). The red curves are the experimental results and the gray curves are a control generated by random shuffling of labels. **e** A scatterplot showing the performance of all 290 models that were generated in detecting mutants in a mixture of wild-type cells and four pathogenic mutants. Each point represents a single model and its position is determined by its ability to distinguish cells in the labeled control wells and cells in the mixed wells. The vertical and horizontal lines represent the AUCs of the model that was used to choose cells for isolation. **f** A histogram of AUCs for models detecting mutants in a mixture of wild-type cells and four pathogenic mutants. A histogram of AUCs generated from randomly assigned models is shown as a comparison.

With the knowledge of each cell’s genotype (*n*_WT_ = 159, *n*_L76P_ = 61, *n*_R94Q_ = 74, *n*_P251A_ = 41, *n*_R280H_ = 6), we found the total accuracy of our on-the-fly predictions to be 72.4% (50:50 = 75.7%, and 10:90 = 64.5%). Therefore, when training with only WT vs. a mixture of *MFN2* mutants, we could predict that individual cells were mutant from an admixture in which we were completely blind to the real genotype using only the subtle mitochondrial phenotype (Fig. 2a, b). As expected, when trying to identify mutants that were the vast minority (in the 10:90 admixture), the model does correctly identify true pathogenic mutant cells, but a number of cells predicted as pathogenic were truly WT. Figure 2c shows the further breakdown of all unlabeled cells by specific mutant and by whether the model correctly identified them as pathogenic mutants (per-mutant metrics are not available in this experiment since we did not train on individual classes of mutants). The mutations most successfully recovered were R94Q and L76P, agreeing with the previous data showing those mutants as having a more severe phenotype (Supplementary Fig. 1). We recovered a similar number of false negatives of the P251A and R280H mutants, despite fewer overall numbers of each mutant, indicating that these mutants likely have a weaker phenotype—leading to a less confident prediction and exclusion from the list of cells to isolate—rather than less penetrance. To check the model quality, we evaluated the receiver operating characteristic (ROC) curves and the resulting area under the curve (AUC) Fig. 2d. The curves in red show the ROC of the model—for the cells picked from labeled (AUC = 0.94) and unlabeled (AUC = 0.74) populations. The AUC for the labeled cells represents the theoretical maximum AUC that we can obtain when applying the model to the unlabeled cells.

We also took a larger view and assessed the quality of *all* models generated (290 in total), not just the one used to select cells for isolation. Figure 2e shows a scatterplot of AUCs (labeled populations vs. unlabeled populations) and Fig. 2f shows the AUC distribution of all models applied to unlabeled populations. Importantly, the majority of models had the discriminatory ability. This entire experiment was repeated successfully using a mixture of all six mutants, and histograms and scatterplots of the models generated are shown in Supplementary Fig. 6.

### Multiple mitochondrial features enhance prediction of *MFN2* mutants

**Fig. 3:**
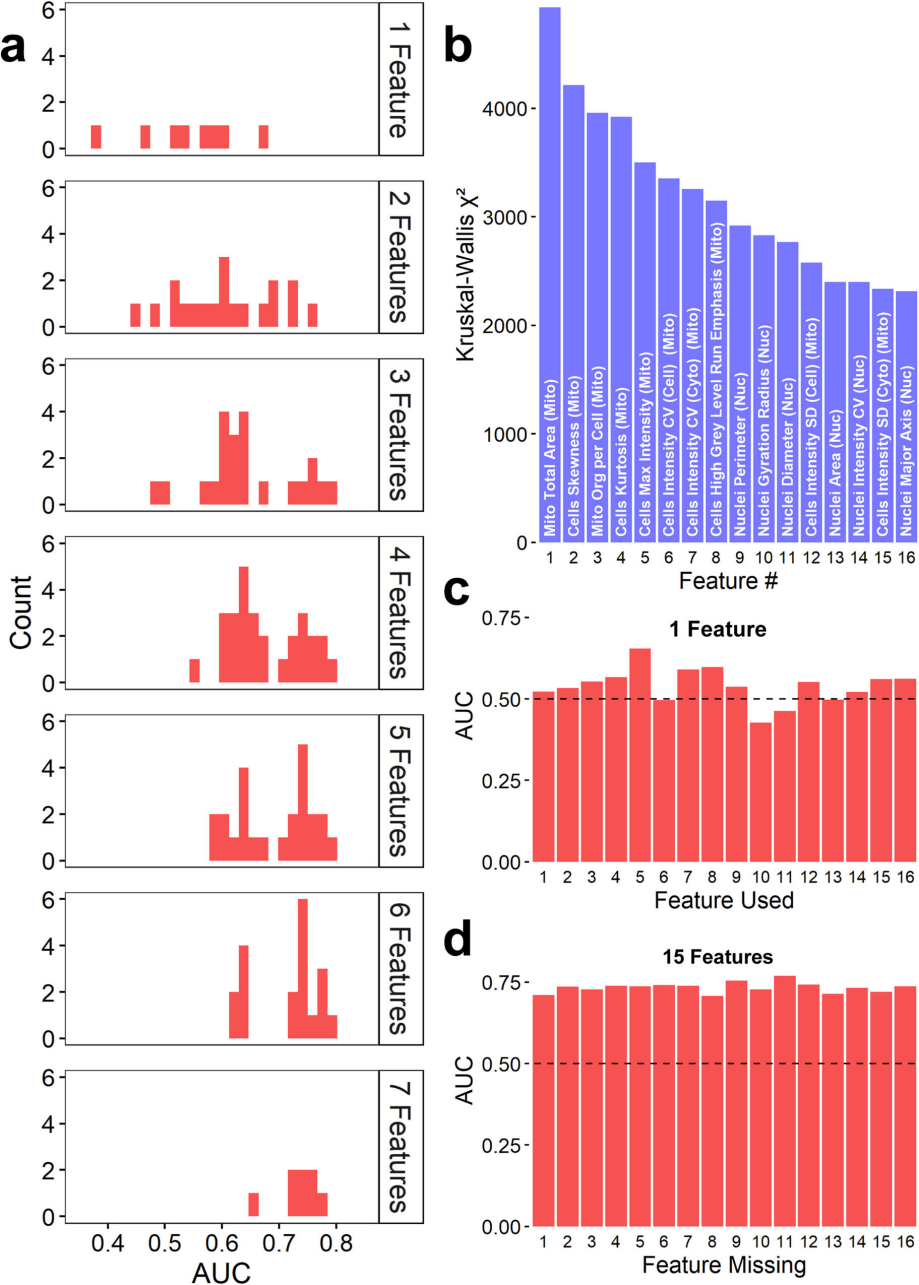
*MFN2* Genotype Prediction does not depend on specific features. **a** Histograms of AUCs resulting from models built from combinations of a set of 8 features used when picking, arranged in panels split by the number of features used in each subset. **b** Bar chart of the Kruskall–Wallis *χ*^2^ values for the 16 features with the highest values. “Mito” and “Nuc” indicate features measured from mitochondrial and nuclear stains, respectively. **c** Bar chart of the AUCs resulting from models built out of each individual feature. Note that the most significant feature was unable to produce a good model alone. **d** Bar charts of the AUCs resulting from models built using all but one feature (leaving 15 features). All AUCs listed indicate the performance of the model trained from labeled data on their ability to predict *unlabeled* cells in admixed conditions.

Multi-feature models were necessary for accurate predictions, warranting exploration into the importance of individual features and the performance impact associated with them. Using data from the experiment containing wild-type *MFN2* cDNA and four pathogenic mutants, we generated additional models by varying the number of features used, sampling from the eight features in the pick model (Supplementary Table 1). Histograms showing the distributions of the AUCs, separated by the number of features are shown in Fig. 3a. When more features are added, the models can better distinguish between the populations of cells. We also examined the importance of individual features in building models (Fig. 3b–d). We used the original feature data (Supplementary Fig. 1) and generated Kruskal-Wallis *χ*^2^ for each feature between the wild-type and pathogenic mutant cells from labeled populations. A ranking of the 16 features associated with the highest *χ*^2^ are shown in Fig. 3b. We compared the resulting AUCs from models built off each specific feature alone (Fig. 3c), and to the resulting AUCs from models built from the 16 features minus one (Fig. 3d). Importantly, we find that single features *are not* the key to discriminating these clinically relevant pathogenic *MFN2* point mutants. Instead, small numbers of relevant features can inform a useful model.

### Improved recovery of weak pathogenic *MFN2 mutants*

**Fig. 4:**
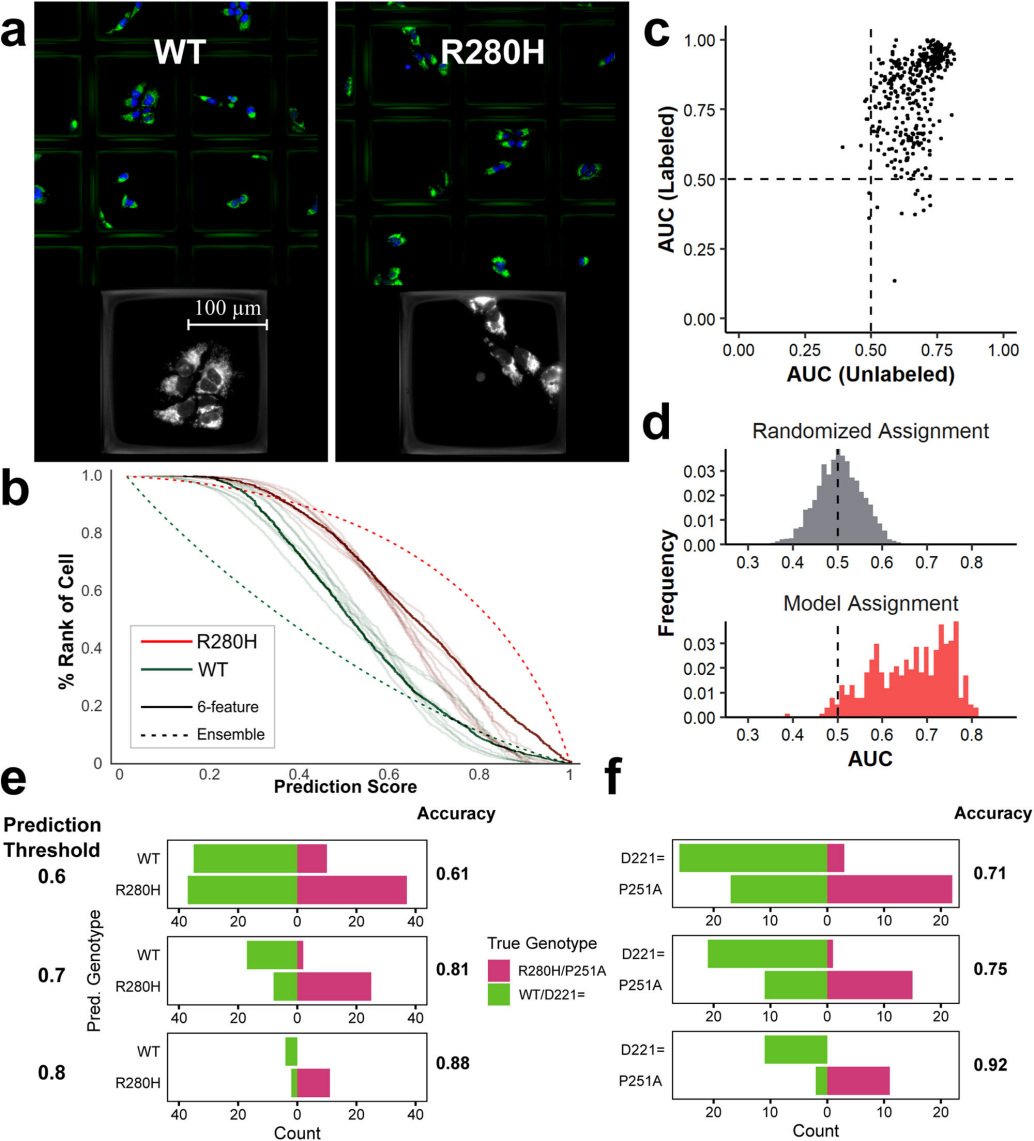
Raft-seq can predict *MFN2* WT and *MFN2* R280H mutant cells which have nearly undetectable phenotypic differences. **a** Images of the R280H mutant and *MFN2* WT cell line in the microraft plate. **b** Ranked histogram of the prediction scores for several models predicting the genotype from the R280H experiment. Each curve shows the distribution of prediction scores for a particular labeled genotype (WT or R280H) where a score of 0 means the model is confident that a particular cell is WT and a score of 1 means the model has a strong prediction of R280H. 8 different 6-feature models are shown. The dashed lines indicate an ensemble model. **c** Scatterplot showing the performance of all 433 models in detecting mutants in a mixture of wild-type cells and the R280H mutant. **d** A histogram of AUCs for models detecting mutants in a mixture of wild-type cells and the R280H mutant. A histogram of AUCs generated from randomly assigned models for comparison. **e**, **f** Accuracies and confusion bar charts of the predictions from the unlabeled wells, when only picking cells with prediction scores ≥0.9 (≤0.1), ≥0.8 (≤0.2), and ≥0.7 (≤0.3). **e** uses the model to predict R280H against WT *MFN2*, while **f** uses the model to predict P251A against D221=.

Our pipeline can also recognize relatively weak phenotypes. Of the six pathogenic mutants, the R280H and P251A appear most like benign mutants (Supplementary Fig. 1c). Despite the subtle phenotype, we recovered R280H cells in the experiment above (Fig. 3b), though comparably fewer than cells with other mutations. Given those results, we explored the ability of Raft-Seq to isolate cells with the R280H mutation compared against *MFN2* wild-type. Figure 4a shows images of R280H mutation-harboring cells in the microraft plate. As a baseline measurement against more conventional methods of cell separation, we ran separate samples of a *MFN2* WT and R280H mutant cell lines each stained with MitoTracker through flow cytometry (Supplementary Fig. 7a). While there is variation between the two in the MitoTracker intensity, there is too much overlap to separate a mixture (Supplementary Fig. 7b AUC = 0.6).

We then followed the Raft-Seq process described above, replacing the mixture of *MFN2* mutants with just R280H mutant cells, and therefore training the models directly on the weaker phenotype. Additionally, the experiment was done with different culture densities and cross-compared to confirm that culture conditions had no “residual phenotype” that impacted the model’s prediction. When we examined the predictions from individual models, it was apparent that many cells were confidently scored as being WT or R280H, while others were less confidently scored by the models, and were therefore not selected for picking (Fig. 4b). Figure 4c, d show histogram and scatterplots for the resulting models in this experiment, demonstrating that Raft-Seq discriminated between the R280H mutant and the *MFN2* wild-type (AUC of picked model 0.72, best models >0.8, *n*_WT_ = 265, *n*_R280H_ = 205). We also ran a similar experiment comparing the P251A pathogenic mutant cell line—the other pathogenic mutation with a weaker phenotype—to the D221 = benign cell line. Cells containing the synonymous substitution D221= act as an alternative control. Supplementary Fig. 8 shows that the resulting model, when applied to a mixture of the cell lines, was able to discriminate between the two (AUC = 0.8, *n*_D221_ = 103, *n*_P251A_ = 65). The AUCs mentioned above apply to all the unlabeled cells that were physically picked. If we limit our results to cells whose class the model is increasingly confident about, then the accuracy of the model gets increasingly better (Fig. 4e, f). For cells that the model is at least 80% confident about (prediction score ≥0.8 or ≤0.2), we get high AUCs and accuracies in both the R280H/WT and P251A/D221 = experiments (AUC = 0.94,0.98 Acc = 0.88,0.92 *n* = 17,24).

We have shown that Raft-Seq can accurately predict genotypes from strong mitochondrial point mutants as well as weak mutants in *MFN2* and predict them as part of a mixed culture where there was no a priori knowledge of each cell’s genotype. The model’s predictions were realistically tested by isolating single cells and genotyping them to reveal the method’s accuracy.

### Mutational scanning of *MFN2* VUS with Raft-seq

While the previous experiments were done by over-expressing a mutant *MFN2* cDNA, we also sought to study editing of an endogenous gene. As a proof-of concept for endogenous mutations, we first used an existing U2OS line with mutations in the mitochondrial primase *PRIMPOL*^41^ and found that it alters mitochondrial morphology (Supplementary Fig. 9). We then followed the Raft-Seq process described above and verified that the platform performs well for an endogenous genetic perturbation (AUC 0.87). We next tested Raft-Seq against a series of (mostly VUS—variants of uncertain significance) mutations in *MFN2*. We constructed a CRISPR-Cas9 gRNA library (Supplementary Data 1) targeting all 18 exonic regions of *MFN2* (CRISPR tiling^42^) on sites near known ClinVar variants (Supplementary Fig. 10) with 357 gRNAs. The library was delivered by lentivirus to U2OS cells with a dox-inducible Cas9 construct, and the cell population was split in two, with only half receiving doxycycline. DNA samples were collected at intervals over a 10-day period for survival screening^43^ which revealed no significant representation differences (Supplementary Fig. 11)—so while mitochondrial changes are observed, those do not result in large viability shifts.

**Fig. 5:**
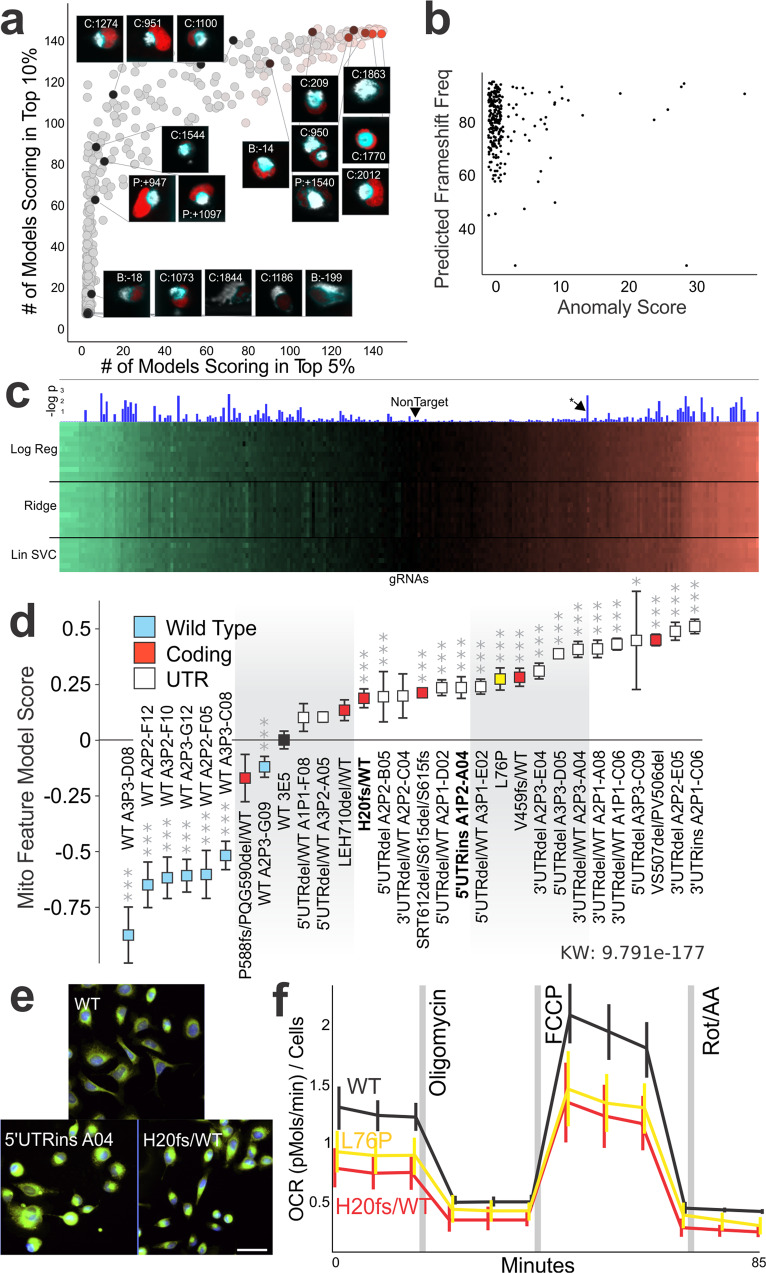
Endogenous scanning mutations in *MFN2* simulate variants of uncertain significance. **a** Scatterplot of genotyped cells. *x* and *y* axes are the number of models placing that cell in the top 5% or 10% of all cells, respectively. A subset of cells are shown with an inset of the fluorescent image taken during the screen and the gRNA present within the cell (labeled by the nucleotide position before the start codon [B], within the coding region [C], or after the stop codon [P], also see Supplementary Data 1). **b** Scatterplot of predicted frameshift frequency vs. mitochondrial anomaly score for all the gRNAs recovered from the screen. **c** Screening results from 357 gRNAs across *MFN2*. The heatmap has 3 sections, each showing half a dozen examples of models constructed with logistic regression, ridge classifiers, or linear SVMs. The color indicates the prediction score of the model where green means a low value (WT-like) and red means a high value (mutant-*MFN2* like). Significance of the gRNA is indicated by the height of the blue bar. **d** Established isogenic lines of U2OS cells with the specific *MFN2* variant(s) rank ordered based on their phenotypes. More “WT-like” phenotypes (likely benign) are shown to the left while more “mutant-like” phenotypes (likely pathogenic) are shown on the right. Bars show average with 95% confidence intervals (*n* = 18–32 well/plate replicates, L76P mutant is shown in yellow and the parental control clone WT 3E5 is shown in black. *p < 0.05, **p < 0.01, ***p < 0.001). **e** Example images of isogenic U2OS cells with either a control gRNA that did not cut, 5’UTRins A04 isogenic line, or H20fs/WT isogenic line. The frameshift-mutant (H20fs/WT) has similar mitochondrial aggregation to the L76P mutant (Fig. 1), while the UTR mutant has a distinct but subtle mitochondrial morphology. Scale bar 50 µm. **f** Metabolic analysis of OCR (oxygen consumption rate) for WT, L76P, and the H20fs/WT lines. OCR is measured in four phases, first basal, then mitochondrial toxins Oligomycin (ATP synthase inhibitor), FCCP (uncoupler), and Rotenone (electron transport inhibitor). OCR is normalized by nuclei count. The asterisk in **c** are V459fs/WT cells spiked into the screen.

Then in a Raft-Seq screening experiment, 68,348 cells were imaged, and mitochondrial anomaly detection models were trained on the feature data of −Dox (no Cas9) cells and deployed to the data of +Dox (Cas9 induced) cells to infer which cells had abnormal mitochondrial phenotypes. Unlike previous modeling techniques, training data of known mutants was not included (necessitating the use of anomaly detection). The most highly anomalous cells had some feature similarity to the strong pathogenic mutants (Supplementary Fig. 12). We selected 1659 cells for isolation, which we then genotyped to identify the gRNA for each cell (Fig. 5a). The result is a rich dataset where each of the single cells are measured for phenotypic features (from confocal imaging) *and* the corresponding gRNA. Since individual gRNAs have different efficiency, we presumed that frameshift edits to *MFN2* would be more likely to result in the strongest phenotypes. We compared each cell’s mitochondrial anomaly score to the probability of the gRNA inducing a frameshift mutation (calculated with Indelphi^44^). Indelphi only yields a prediction, and each single cell with a gRNA may have different genomic consequences of the Cas9 cut. We found that nearly all gRNAs with a high anomaly score also had a high predicted frameshift mutation rate (Fig. 5b, *p* value at 3 frameshift thresholds: 90% = 0.0013 [*n* 42], 80% = 0.05 [*n* 111], 70% = 0.37 [*n* 39]).

We next undertook a broader screening experiment with Raft-Seq on the scanning mutagenesis library of MFN2 (357 gRNAs). After imaging 76,574 cells across five replicate experiments and selecting 1,222 rafts to single-cell sequence for gRNA identification, modeling was performed. 479 supervised binary classifiers were trained on a subset of 11,876 cells with known genotype. Each model then inferred a prediction score on all 76,574 cells. An overview of the screening result (Fig. 5c) shows that while the models produce different scores, they are generally in agreement about whether the hit was more WT-like (left green side of the graph) or *MFN2* mutant-like (right red side). This screening set also provided for metrics of measuring reproducibility, and we found that 74.7% gRNAs (which could be measured for reproducibility across replicate experiments) were reproducible in the screen (Supplementary Fig. 13).

Next, we utilized some of the core components of the Raft-Seq pipeline to isolate perturbed single cells from an *MFN2* gRNA-infected library for downstream analysis and experiments. We captured the cells intact and alive into tissue culture plates and grew them clonally as isogenic lines. This allowed enough genomic material to do two rounds of sequencing—first to find the gRNA present in each cell and second to examine the gRNA target site—and use the cells for downstream analyses, something that has not been previously done in other imaging-based perturbation screens (could theoretically be done with Craft-ID^30^). Most of the isogenic lines with Cas9 edits were in the UTRs, while six affected the protein (Supplementary Table 2). For example, the isogenic H20fs/WT had a gRNA targeted to the first translated exon, which resulted in a 7 bp deletion, then a frameshift. We analyzed the isogenic cells (arrayed) to quantitatively assess the level of mitochondrial abnormality (Fig. 5d). Three dozen of the individual mitochondrial or TMRM features (Supplementary Table 3), as well as all multi-feature models, showed significant differences between the set of WT clones and the mutated clones (Supplementary Fig. 14).

We performed additional validation experiments on two of the clones, one UTR mutant and one coding mutant (Fig. 5e). We found that these isogenic endogenous mutations were separable from a WT clone in our Raft-Seq mix assay (AUC = 0.90, Supplementary Fig. 15). We also analyzed the set of *MFN2* isogenic lines using the Seahorse XF assay, which analyzes key metabolic processes reflective of mitochondrial health (Supplementary Table 4). Both the H20fs/WT and the L76P mutants had a reduced basal oxygen consumption rate when compared to WT, indicating a mitochondrial deficit from the mutations (Fig. 5f). Overall, we showed pooled cellular screening with Raft-Seq and its ability to generate isogenic lines with mutant phenotypes, including novel mutations that have not previously been studied.

## Discussion

We have developed a method, Raft-Seq, to efficiently screen many genetic variants based on their impact to a cell’s phenotype. We have shown that our method can effectively discriminate between wild-type cells and cells containing different pathogenic point mutations of the *MFN2* and *PRIMPOL* genes. Though we utilize a particular individual model for the selection of cells for isolation, we found that most of the models that we generate can identify pathogenic mutant cells. Since our feature selection process is only mildly dictated by the actual phenotype that we are looking for, our ability to predict a cell’s genotype comes from quickly generating complex computational models. This enables our pipeline to exploit more phenotypes than the scientific literature is currently familiar with, therefore making almost any gene in the genome amenable to this functional screening and thus enabling the re-cataloging of VUS as benign or pathogenic.

We developed Raft-Seq primarily as a screening platform to work in concert with Deep Mutational Scanning^45^, a method for creating a library of every single possible mutation in a gene. Our results show that our screen can correctly identify pathogenic variants, since we were able to recover pathogenic *MFN2* mutants from a mixture with wild-type cells. We were also able to see how Raft-Seq would perform in the discovery of novel variants and morphology. In a small, pooled screening experiment, we examined 357 different gRNAs against *MFN2*, and had reproducibility ~75% amongst experimental replicates. Our anomaly detection models were able to identify similar mitochondrial features to the pathogenic variant cells, and this anomalous morphology correlated with a high predicted frameshift frequency from the gRNA contained in the cell. Lastly, we used Raft-Seq to generate isogenic cell lines directly from the primary screen. We analyzed these clonal/isogenic lines to find consistent mitochondrial phenotypes and blunted metabolic responses. This process presents a large gain over other phenotyping screens, since we can generate cell lines that can be more deeply sequenced and assayed, while simultaneously tracking each line to the specific image and features from which it was selected in the original screen.

Since Raft-Seq isolates cells for sequencing individually rather than in pools, we have a few advantages over other platforms. For one, we are able to find the specific genotype of each isolated cell, rather than having to perform batch measurements, meaning that we can identify effects of combinations of perturbations. We can also have as many categories in our machine learning models as cells, though using more than a handful of features causes the modeling and analysis to deteriorate, likely due to overfitting (but is countered by higher n). Another advantage of this approach is the flexibility that comes from using machine learning to identify phenotypes. Since we can easily combine features for more accuracy, we can potentially screen for any visible phenotype, given a strong enough signal provided by staining or other fluorescence. Unsupervised learning methods, like clustering, are also possible and allow for a simpler setup since no labeled wells would be needed.

From the mutant mixing experiments, there are some factors to consider. For example, based on the accuracy difference between the two unlabeled wells housing cells at two different ratios of WT:pathogenic, our ability to recover pathogenic variants appears to decrease as the proportion of pathogenic variants present decreases (low ‘hit’ rate). This indicates that such a screen would perform significantly better on genes for which mutations are more likely to result in a pathogenic variant. This limitation can be potentially countered with a higher number of cells if studying mutant phenotypes that are distinct from wild-type. It is difficult to examine phenotypes that have highly overlapping distributions between wild-type and mutant for any screening method that classifies phenotype on a single-cell level, such as Raft-Seq. Second, we were able to identify the mutants with more extreme phenotypes (L76P, R94Q) more frequently than other mutants with subtler phenotypes, meaning that a scaled-up screen would show similar results, with more extreme variants being overrepresented. However, because we were able to identify the weaker mutants (R280H, P251A) with relative ease when they were not mixed with any other mutants, we can assume that the overrepresentation of specific variants is not due to the absolute strength of the resulting phenotype. Instead, we assume it is due to the relative strength when compared to other variants.

Several other technologies can perform pooled screening for imageable phenotypes^46^, and these screening-imaging systems are listed in Table 1 for comparison. The quality of imaging varies by system (Table 1a). Arrayed screening (where each condition is separated by well) utilizes confocal microscopy without constraint. Raft-Seq and CRaft-ID also employ high-content imaging, but optical *working distance* is increased because of the raft design, and thickness variations cause occasional focus failures. ISS and photo activation (PA) are restricted in imaging by choice of dyes that do not conflict with the sequencing chemistry or photoactivatable chemical, respectively. ISS and PA can overcome these conflicts with custom probes and careful titration. The techniques vary greatly on how individual cells are “IDed” for the perturbation that was made (Table 1b). Both raft platforms rely on single raft collection to isolate cells while PA collects cells through FACS sorting of labeled cells. Arrayed screening and ISS do not capture the cell at all. The ability to collect cells physically opens other possibilities, but the state of the cells vary (Table 1c, d). The raft platforms can both collect fixed or high-viability live cells, but Raft-Seq is tuned towards single cells and CRaft-ID is optimized for colonies. ISS can image and sequence at single-cell resolution, but the cells are rendered non-viable in the process. PA collects *bulk* cells, which removes the 1:1 relationship between an image and a specific sequence. The sorting methods subject the cells to more pressure and stress than the raft methods, which instead leave the cells adhered to the plate while the transfer occurs (critical for delicate cells like neurons). The collection of viable cells allows for the production of isogenic cell lines from single mutants (for example).

**Table 1:** Cell screening-imaging platforms.

Comparison criteria	Arrayed screening^8,48^	In situ sequencing^24,25^	Photo-activation^18,20,27,49^	CRaft-ID^30^	Raft-seq^(this paper)^
a. Imaging constraints	No limitations	Impacted by seq chemistry	Limited by # of usable channels	Optics impacted by raft	Optics impacted by raft
b. How cells are Collected	NA	NA	FACS	Raft picking	Raft picking
c. Genotype resolution	Bulk	Single cell	Bulk	Colony	Single cell
d. Live cell collection	No	No	Medium viability	Possible	Yes
e. Genotyping method	NA	Specialized	Bulk NGS	Colony NGS	Single-cell NGS
f. Pooled scalability	Not pooled	Highest	Yes	Yes	Yes
g. Bottleneck	Physical plates	ISS Technology	Photo activation	Raft picking	Raft picking

In terms of genotyping (Table 1e), Raft-Seq, Craft-ID, and PA utilize standard Illumina sequencing (although Raft-Seq and Craft-ID need more preamplification due to the small starting amount). ISS originally required a custom sequencing microscope/rig, but there are now several commercial options. While the read length is limited because of the in situ reaction parameters, it is still long enough for a barcode which provides the identity of the perturbation for a screen with less than a thousand perturbations.

Except for Arrayed screening, the other methods listed start with all the cells/perturbations in a *pool* (Table 1f, g), making them highly scalable. As compared to a PA screen^20^, the throughput of Raft-Seq and a PA method is similar, as is the number of cells captured over a subset of the experiment with a scale of about a thousand perturbations. Raft-Seq has the capability to image 500 K single cells per day. The rate-limiting step is physically picking the single cells with the Cell Microsystems instrument (limited to around 3 K per day). Since so many more cells can be imaged than picked, we can increase the pool of isolation candidates, allowing cells to be selected with higher confidence. Translating those numbers into a full screen then depends on the strength of the phenotype and the rate of hits in the screening set.

There are two steps in Raft-Seq that are done manually but will soon be automated. The first is image quality control which can be automated by a convolutional neural network, similar to the previous microraft experiments^30^. The second is model selection, which can be automated by choosing among several model performance metrics on the labeled data.

Since the raft identifies the location of the cell stably over time, we can do on-the-fly training from the entire experiment, then go back and select cells to pick. We have also found that Raft-Seq can be used to apply past training (labeled) data to future experiments. A normalization schema allows for accretion of previous training data to utilize it in future models (allowing a gain of accuracy in identifying specific phenotypes over time).

We are optimistic that with increased scale, Raft-Seq can be used to sensitively find variants across a wide breadth of perturbed cellular phenotypes. An increase in scale would make the models more accurate, both by providing more training data and by identifying more cells that are confidently classified by the model. Altogether, we have shown that using various cell lines we can deploy flexible machine learning from data within an experiment (on-the-fly) or from previous experiments (pre-trained) to select clinically relevant point mutations in a screening setting.

## Methods

### Cell culture and transfection

Human osteosarcoma (U2OS, ATCC HTB-96) cell lines were maintained in McCoy’s 5 A Modified Medium (16600082, Gibco, Gaithersburg, MD, USA) supplemented with 10% fetal bovine serum (FBS) (16000044, Gibco). Human embryonic kidney (HEK) 293 T cells (CRL-11268, ATCC) were cultured in Dulbecco’s Modified Eagle’s Medium (11965-092, Gibco, Gaithersburg, MD, USA**)** supplemented with 10% FBS (16000044, Gibco, Gaithersburg, MD, USA), 1% Penicillin-Streptomycin (15140122, Gibco) and 1% non-essential amino acids (11140050, Gibco).

All cell lines were maintained in T75 tissue culture flasks in an incubator at 37 °C, 5% CO_2_ and they were observed daily for growth and overall health. Once confluent, cells were passaged using 0.25% Trypsin-EDTA 1× (25200056 Gibco, Gaithersburg, MD, USA) at a sub-cultivation ratio of 1:10. Live-cell counting was performed with the BioRad TC20 automated cell counter. Centrifugation of cell cultures was performed at 1200 rpm for 3 min. Lentiviral infection was performed in T75 flasks when cells were 85% confluent. STR profiling, to confirm cell type, was performed using NGS-based analysis by the Genome Engineering and Stem Cell Center (GESC) at Washington University in St. Louis. Testing for mycoplasma was performed bi-annually. For all experiments in this paper, either 100 × 100 or 200 × 200 micron quad reservoir plates containing 48,000 (12,000 cells per quad) and 36,000 cells (9000 cells per quad), respectively were used. Prior to plating, microraft plates were prepared by rinsing with 1 mL PBS three times with 3-min incubation periods. Cells were added in 200 µl media to aid in distribution, then plated and incubated overnight (14–16 h).

### Virus production and *MFN2* single mutant line creation

*MFN2* lentiviral expression plasmids were cloned into the CCIV lentiviral plasmid with a GFP marker^37^. In preparation for lentiviral packaging, 8.0 × 10^5^ HEK293T cells were plated into each well of a six-well plate and incubated at 37 °C overnight. The cells were then transfected with TransIT Lenti-transfection reagent (MIR 6600, Mirus Bio, Madison, WI, USA) using an envelope plasmid (pVSVg: Addgene plasmid # 8454), a packaging plasmid (psPAX2: Addgene plasmid # 12260), and each individual *MFN2* expression plasmid in a mass ratio of 0.5/1/0.5 respectively for a total of 2 µg. After 48 h, media was collected, centrifuged, and sterile-filtered before being concentrated (Lenti-X Concentrator 631232 Takara Bio, Kusatsu, Shiga, Japan). The concentrated virus was resuspended in 200 µL 1× PBS per well, collected, and stored at −80 °C.

To create stable *MFN2*-mutant expressing lines, T75 flasks containing 6 million U2OS cells were infected with 70 µL of concentrated lentivirus at an MOI > 1 and polybrene was added (NC9840454 Santa Cruz Biotechnology, Texas) at a final concentration of 10 µg/mL. They were then incubated for 24 h, after which the virus-containing media was removed and replaced with fresh, virus-free media. Cells were taken to the Washington University Siteman Flow Core for fluorescent sorting on the Sony Synergy, 100-micron sorter. Cells were sorted based on viability and GFP expression (since no puromycin selection was performed, the fluorescent signal from the GFP in the *MFN2* plasmid was used to determine transgene expression). GFP expression levels were compared within and across generated cell lines to ensure population purity and comparable fluorescent expression levels. The PRIMPOL KO U2OS cell line was received from the Vindigni lab and was produced by the GESC.

### CRISPR/Cas9 gRNA library infection and induction

A dox-inducible Cas9 (iCas9) U2OS cell line was generated via CRISPR-mediated homology directed repair. The Cas9 protein, gRNA, and donor construct were introduced via nucleofection. Isogenic iCas9 clones were isolated using the Cell MicroSystems CellRaft AIR System and then propagated for further experiments. Presence of the construct was validated via junction PCR^47^ prior to propagation. Puromycin-resistant *MFN2* scanning gRNA libraries were generated and cloned by the Washington University GESC. Lentivirus was produced (see Virus Production above) and used to infect iCas9 U2OS cells at an MOI of <0.2 followed by 8 µg/mL puromycin selection for seven days. The cells were then allowed to grow in fresh media. At 60–70% confluency, Doxycycline (Cat#: D9891-1G, Millipore Sigma) was added at a final concentration of 2 µg/mL. The cells were incubated at 37 °C for 48–60 h before proceeding with staining and imaging.

### Staining and microscopy

The following vital dyes were used; DNA labeling/nuclei (Hoechst, Thermo Fisher H3570), mitochondria (MitoTracker Deep Red, Thermo Fisher M2246), and mitochondrial membrane potential (Tetramethyl Rhodamine methyl ester TMRM, Thermo Fisher I34361). MitoTracker and TMRM were incubated for 40 min at concentrations of 0.5 and 0.1 µM, respectively. Hoechst was incubated for 15 min at a concentration of 10 µg/mL (16.2 µM). Each plate was rinsed twice with culture media prior to imaging. Images were captured using a 20 × 0.45 NA objective in the Cytiva INCell 6500HS Confocal microscope. Exposure times for Hoechst (405 nm) and TMRM (561 nm) averaged 0.15 seconds while MitoTracker Deep Red (642 nm) averaged 0.05 seconds. Confocality was used in the 405 and 642 wavelengths to decrease the background fluorescence of the CytoSort raft plate. Each field-of-view overlapped by 12% of their area. Imaging settings were held constant throughout the course of an experiment. Following imaging, an extra 500 µL of cell culture media was added to the CytoSort raft plate (additional liquid helps the CellRaft AIR System isolate microrafts).

### Image analysis and quality control

Image tracing and feature extraction was performed using Cytiva’s INCarta software. Mitochondrial puncta were identified (within 20 µm of the nuclei using the ‘networks’ algorithm) and quantified for each cell as were a set of texture features. Raft coordinates were recorded for each cell (using FIVTools/ CalCheck, included in the GitLab repository). Images were also curated semi-manually (via FIVTools/ CalCheck) to ensure that out-of-focus images were excluded. The cell feature dataset was joined with the image quality data and raft position mapping data described above by custom software (via FIVTools/ main window). Post tracing quality control was performed with each dataset in Tibco Spotfire Analyst. First, aberrant tracing artifacts were excluded based on nuclear area, nuclear form factor, and proximity to the raft’s edge. Next, non-nuclear debris and dead nuclei were excluded by gating on nuclear area, intensity, and cell intensity. Rafts with too many cells (>6) or a fiduciary marker were excluded. The resulting dataset typically contains 170 cell body, nuclei, or mitochondrial measurements. This filtered set of cells is normalized by plate and used for the machine learning models downstream.

### Machine learning and model generation

After exporting the quality-controlled cell-based feature table, we built multiple supervised binary classification models that used cell-specific phenotype measurements as explanatory variables. The response variable defined each cell as known “**WT**” or pathogenic mutant “**MU**”. Although the “MU” label was applied indiscriminately to various cells, these cells manifest a variety of perturbed phenotypes; therefore, the machine learning models’ understanding of what constitutes a perturbed phenotype was limited to those phenotypes manifested in the training set. There may be other legitimate (but quite dissimilar to the positive controls) perturbations in the cell population to which our binary classification models were blind. This is a limitation of a binary class setup and is one advantage of anomaly detection models.

### Statistics and reproducibility

For clarity, when we refer to ‘genotype’ we mean the specific point mutation present (R280H, WT, H20fs, etc). If we refer to ‘genotype class’, these are WT, Benign, or Pathogenic (aka what is designated by the model). ‘Labeled Cell Population’ are the cells used for training the models and ‘unlabeled cell population’ are admixtures of labeled cells (like benign + pathogenic) or in screening, where the researchers are completely blind to the identity of individual cells. Prior to training, all cell feature vectors were normalized, and if the proportion of WT to pathogenic labels was unbalanced, they underwent synthetic minority oversampling. Feature selection was accomplished by several techniques, including setting a threshold minimum for variance within each feature vector, setting a threshold maximum for correlation between features, randomly selecting features (usually numbering between 2 and 20), selecting only the k highest scoring features according to their ANOVA F-value, and selecting from feature importance according to a Linear Support Vector Machine with L1 regularization.

The models were trained and used to select cells within the same day. A variety of machine learning platforms (Microsoft AzureML Studio, Scikit Learn, and H2O.ai.) and algorithms were employed to predict a cell’s unknown genotype class. The details on which models, features, and hyperparameters are listed in Supplementary Note 1. Models were evaluated by their AUC, Matthew’s correlation coefficient and their ability to distinguish between labeled populations that were withheld during training. Based on model performance on the testing dataset, a trained model was selected and used to infer prediction probabilities on the unlabeled cell populations. While the genotype of the cells in the unlabeled population was unknown, the expected proportion of WT to perturbed cells was known (a meta-feature). The final evaluation of our model was to plot a ranked histogram that included these unlabeled populations for which the underlying distribution of WT to perturbed cells was known and determine if the model produced prediction probabilities that accord with that distribution (This can be seen in Supplementary Fig. 9b). This meta-feature was one of the strongest protections against overfitting. We then selected cells with the highest and lowest inferred prediction probabilities from our selected model to generate the list of cells (and therefore rafts) to be picked. Details on the statistics are listed throughout, and a special section is included on reproducibility which is referenced in Supplementary Fig. 13. Statistical significance of gRNAs of interest was computed using a Benjamini–Hochberg corrected one sample *t* test with a false discovery rate of 0.2.

### Cell capture and DNA extraction

Cells were isolated using the Cell MicroSystems CellRaft AIR System. CytoSort raft plates were received from Cell Microsystems (Durham, North Carolina). Given a list of raft coordinates, the AIR System used a needle to eject each individual raft and transfer the raft to a semi-skirted 96-well PCR plate (1402–9200, USA Scientific) via a magnetic wand. Each well of the PCR plate contained 5 µL extraction buffer (molecular grade water with 10 mM Tris-HCl (pH 8.0), 2 mM EDTA, 200 µg/mL Proteinase K, and 0.2% TritonX-100). Raft isolation was confirmed twice through post-ejection imaging of the raft location and through visual inspection using a Leica S8AP0 dissection scope, after DNA extraction. Genomic DNA was extracted in a thermocycler immediately following raft isolation by incubating at 65 °C for 15 min than at 95 °C for 5 min.

### Single-cell DNA amplification

Amplification of single-cell DNA prior to library preparation consisted of two separate amplifications. An initial preamplification was conducted using extracted DNA with KOD Hot Start DNA Polymerase (71842-4, Millipore Sigma, Burlington, MA, USA) according to manufacturer’s instructions using all 5 µL of extracted DNA in a total reaction volume of 20 µL. Pre-amplified product was processed through an AMPure XP (Catalog: A63882, Beckman Coulter, Brea, CA, USA) bead clean-up according to the manufacturer’s instructions using 10 mM Tris-HCl pH 8.5 as elution buffer. The second amplification used the cleaned template and BioLine MyTaq HS Red Mix 2× (C755G97, Meridian Life Sciences, Memphis, TN, USA), according to the manufacturer’s instructions, including 5% by volume DMSO. Primers in the second amplification contained universal 5’ tags to be compatible with Illumina library preparation (Forward tag: 5′-CACTCTTTCCCTACACGACGCTCTTCCGATCT-3′, Reverse tag: 5′-GTGACTGGAGTTCAGACGTGTGCTCTTCCGATCT-3′).

For the amplification of *MFN2* cDNA, primers amplifying the entire cDNA were used in the first amplification step, followed by multiplexed amplification of two specific regions containing the relevant mutations. Genotyping of the RFP-GFP cells used multiplexed primers that amplified specific regions in both the RFP and GFP regions. All primers are listed below in Table 2.

**Table 2:** Genotyping primers.

Name	Sequence (excluding tags, where necessary)	PCR stage
pMFN2.All.F	GCTCTTCTCTCGATGCAACTCT	1
pMFN2.All.R	GCAGGTACTGGTGTGTGAAC	1
pMFN2.1.F	CACATGGCTGAGGTGAATGC	2
pMFN2.1.R	GCAGGAAGCAATTGGTGGTG	2
pMFN2.2.F	CTCAGAGTCCACCCTGATGC	2
pMFN2.2.R	CACTTGAAAGCCTTCTGCGAG	2
RFP.F	GTTCATGCGCTTCAAGGTGC	1, 2
RFP.R	CAAGTAGTCGGGGATGTCGG	1, 2
GFP.F	TGAAGTTCATCTGCACCACCG	1, 2
GFP.R	TCGCCCTCGAACTTCACCTC	1, 2
PRIMPOL.F	GCAACCCAGTTTTGAAACCA	1, 2
PRIMPOL.R	TCGATGTCCAGCTTTCCTCT	1, 2
gRNA.F	CTTGTGGAAAGGACGAAACACC	1, 2
gRNA.R	TTGTGGATGAATACTGCCATTTGT	1, 2

### Illumina library preparation

These methods are expanded from Connelly et al. and Bell et al. After amplification with universal primers, each plate was amplified with specific forward and reverse Illumina index primers that indicate the PCR plate position and a unique plate ID. PCR amplification was performed with BioLine MyTaq HS Red Mix 2× (C755G97,Meridian Life Sciences, Memphis, TN, USA) according to the manufacturer’s protocol, pooled, and then cleaned using AMPure XP beads (A63882, Beckman Coulter Life Sciences, Indianapolis, IN, USA). DNA was quantitated on a NanoDrop One Spectrophotometer (Thermo Scientific, ND-ONE-W) before being submitted to the Center for Genome Sciences and Systems Biology (Washington University) to generate 2 × 250 reads on the Illumina MiSeq platform.

### Sequencing analysis

Illumina paired reads were demultiplexed by the core facility and FastQ files were returned. The rest of the analysis was performed with laboratory software available on Gitlab (FIVTools/ LA, “Library Aligner”). Reads were joined and trimmed, then aligned with small sequence fragments at the genetic sites of interest containing the sequence to mutant or WT alleles. The result was a ‘counts’ table that gave the number of reads containing each 20-mer for each well. 20-mer search fragments are listed below in Table 3. After accounting for isolation and genomic amplification errors, ~80% of the isolated cells genotypes were captured.

**Table 3:** 20-mer fragments.

Name	20-mer (relevant mutations/deletions bolded)
MFN2_V69	TGGACCCCGTTACCACAGAA
MFN2_V69F	TGGACCCC**T**TTACCACAGAA
MFN2_L76	ACAGGTTCTGGACGTCAAAG
MFN2_L76P	ACAGGTTC**C**GGACGTCAAAG
MFN2_R94	TGCTGGCTCGGAGGCACATG
MFN2_R94Q	TGCTGGCTC**A**GAGGCACATG
MFN2_D221	CTGGATGCTGATGTGTTTGT
MFN2_D221=	CTGGATGCTGA**C**GTGTTTGT
MFN2_P251	CTCTCCCGGCCAAACATCTT
MFN2_P251A	CTCTCCCGG**G**CAAACATCTT
MFN2_R280	CATGGAGCGTTGTACCAGCT
MFN2_R280H	CATGGAGC**A**TTGTACCAGCT
MFN2_W740	AAAGCCGGTTGGTTGGACAG
MFN2_W740S	AAAGCCGGTT**C**GTTGGACAG
RFP_guide	GGCCACGAGTTCGAGATCGA
RFP_control	AAGGTGCGGATGGAGGGCAG
GFP_guide	TGCCCGAAGGCTACGTCCAG
GFP_control	CTACCCCGACCACATGAAGC
PRIMPOL_WT	GATAGCGCTCCAGAGA**C**AAC
PRIMPOL_del	GATAGCGCTCCAGAGAAACA

For *MFN2* cDNA genotyping, each mutation locus was given a %mutant score calculated as the number of mutant reads divided by total number of reads at that locus. Cells were designated as wild-type if no locus had >50% mutant score, otherwise they were designated as a specific mutant based on which locus had the highest mutant score (ambiguous cells were excluded). Lastly, a flat file was exported containing each picked raft and its assigned genotype.

Using our custom software (FIVTools/AUC), we joined the modeling and genotyping flat files to find overall accuracy and generate ROC curves for each model. We also generated ‘noise’ ROC curves by shuffling the assigned genotypes. Prediction scores between 0.4 and 0.6 were filtered out (scores ~0.5 meant the specific model was unable to classify these cells). For the data presented in Fig. 4e, f, this threshold was further adjusted.

### Isogenic line production

For clonal cell growth, single live cells were isolated by the CellRaft AIR System into 96-well tissue culture plates (TPP 92096), containing 200 µl of media per well. As the isogenic lines grew, the entirety of each well was passaged into a plate of larger size (96-well to 24-well to 12-well to 6-well plates from TPP) after reaching ~70% confluency. It took 2–3 weeks to go from single-cell to 70% confluency in the 96-well plate, and during that time wells were checked for contamination and media level every 2 days. After the cells were plated in the 6-well plate, they were grown to 90% confluency and one-third of the cell suspension was taken for genotyping, one third was frozen for long term storage, and the remaining third was kept for downstream experiments. The entire process took ~2.5 months to go from single cells to frozen stocks/genotyping data (with a large fraction of cells not growing enough to go to the next stage). Genomic DNA samples were initially genotyped to determine the gRNA(s) present (as described in the preceding sections). Following identification of specific gRNA(s), primers were designed by identifying regions containing gRNA target sites and finding primers that encompassed those regions (Supplementary Data 1). The genomic DNA samples were then amplified and genotyped a second time using the primer set(s) specific to the target regions in the sample.

### Metabolic analysis

All metabolic analyses were conducted using an Agilent SeahorseXF96 extracellular flux analyzer. Cell culture microplates (Agilent 102601-100) were seeded with 50,000 cells 24 h prior to running the assay. Sensor cartridges (Agilent 102601-100) were hydrated with sterile water and incubated, along with XF calibrant (Agilent 100840-000), in a non-CO_2_ incubator 24 h prior to use. Complete Seahorse assay medium (Agilent 103680-100) was made immediately prior to running the assay according to the manufacturer’s instructions. 160 µL of XF calibrant was added to the entirety of the plate. The cell culture microplate and sensor cartridge were then incubated at 37 °C in a non-CO_2_ incubator. All assays performed used the Seahorse XF Cell Mito Stress Test Kit (Agilent 103015-100) with Oligomycin 1.5 µM, FCCP 1.0 µM, Rotenone/Antimycin A 0.5 µM, compounds were reconstituted and diluted using complete seahorse medium on the day of the assay. Cell number normalization was performed through image-based counting of cells prior to running the assay (using the InCell as described above).

### Cell picking

Each raft has a four-character alphanumeric coordinate (Raft ID). Fiduciary markers located at fixed locations on the raft plate (Supplementary Fig. 16) in conjunction with custom software (FIVTools > Cal Check, Calibration) were used to locate individual rafts for map generation (see “Image Analysis and Quality Control”).

### HEK293 cell nucleofection

SF cell line solution stock was prepared from the Lonza SF Cell line 4D-Nucleofector LV Kit XL (V4LC-2520) kit by combining 82 µL of SF Cell Line Nucleofector^TM^ Solution with 18 µL Supplement 1. 20 µL of stock solution was then combined with 2 µL 1 mg/mL Cas9 protein (QB3 MacroLab) and 2 µL 1 mg/mL gRNA. The complexes were incubated on the benchtop for 10 min. Cell suspensions of 2 × 10^5^ cells were placed in 1.5 mL microcentrifuge tubes and spun down. The supernatant was carefully removed avoiding the cell pellet. The cells were rinsed in 1× PBS and centrifuged a second time. The supernatant was carefully removed, and the cells were resuspended in the final combined SF cell line solution and transferred to a nucleofector cuvette, found in the nucleofector kit. The cuvette was placed in the Lonza 4D-Nucleofector Unit (Lonza AAF-1002X, AAF-1002B) and nucleofected with Pulse code CM130. Nucleofected material was added to a prewarmed six-well plate with 5 mL of DMEM media in each well. Nucleofected cells were incubated for 48 h for recovery. The cell line solution is not healthy for the cells, so the speed is a priority upon resuspension in the cell line solution.

### RFP-GFP cell perturbation and isolation

A HEK293 cell line expressing both RFP and GFP (Gentarget #SC009) was used in this experiment. Cells were then nucleofected with a gRNA targeting GFP (TGCCCGAAGGCTACGTCCAG) or RFP (GGCCACGAGTTCGAGATCGA) and Cas9. All gRNAs were ordered from Synthego. Nucleofection was conducted using a Lonza 4D-Nucleofector Unit. (Lonza AAF-1002X, AAF-1002B). Following the Raft-Seq workflow, cells were imaged and the guide presence was predicted by a combination of RFP and GFP intensity features. Cells were selected and isolated into 96-well plates. Alternatively, cells were sorted using a Sony SH800S cell sorter individually by their RFP and GFP fluorescence. Cells were then genotyped and designated as being given the RFP or GFP guide if the locus that the respective guide targets had been altered.

### Modeling considerations

We implemented several specific design criteria during the experimental setup and model selection. First, the ‘pure’ samples were split into testing wells and training wells so that we could decrease overfitting during model selection. For most of the, the training datasets consisted of 2 of the 3 labeled wells of each type—these two wells came from different plates—making the remaining two labeled wells the “testing sets”. We split the data by well, rather than randomly assigning cells from all pure population wells, to mitigate the influence of batch effects during modeling. The data used for validation is the gRNA identities from the unlabeled cell population (that are completely unknown at the outset). Since training data and testing data technically come from separate samples, we observed skewed distributions from the batch effects, which can present a large and consistent obstacle to cell imaging analysis (Caicedo et al.). In the screening experiments, the training and testing data were derived from a randomized 80–20 split, and batch effects were dealt with through the implementation of a multi-dataset integrating algorithm (harmony). Relatedly, we made sure not to create any artificial batch effects generated by inconsistencies in the screening process. All stains used were prepared as a single batch to be used across plates. All imaging and feature extraction settings were kept constant across each plate in a screening experiment.

Another aspect of model design that we considered is the exclusion of “leaky” variables, or variables that happen to correlate with the data labels. Since our labeled data existed on specific positions of the plate, including a variable that is effectively a proxy for cell position would render the model useless on unlabeled data. We found that several variables that are in the standard output of INCarta feature extraction are leaky, such as features measured on a global basis, and we excluded them before starting the modeling process.

We also faced a time constraint in our modeling pipeline, caused by the necessity to keep cell locations constant between imaging and isolation. Though we could fix the cells, this would limit our ability to perform Raft-Seq for the isolation of live cells. Optimally, we completed feature selection, modeling, and raft selection within 6 hours of imaging, and continued on to isolation.

### Reporting summary

Further information on research design is available in the Nature Research Reporting Summary linked to this article.

## Supplementary information

Supplementary Information

Description of Additional Supplementary Files

Supplementary Data 1

Supplementary Data 2

Supplementary Data 3

Supplementary Data 4

Reporting summary

## Data Availability

Figure source data is provided in Supplementary Data 2–4.

## Code availability

All custom software can be found at https://gitlab.com/buchserlab/FIVTools. To investigate the version used in the manuscript instead of the newest version, pull the commit with FIVTools SHA b56214c98ea0a0df788ce8b75a75850cc1f29d59, and Jupyter SHA 5000c85f40cdd88c9615fa57d533d147a8266dfa.
