# Supplementary material for: Pooled image-base screening of mitochondria with microraft isolation distinguishes pathogenic mitofusin 2 mutations
Source: Commun Biol. 2022 Oct 25;5:1128. doi: 10.1038/s42003-022-04089-y (PMC9596453; doi:10.1038/s42003-022-04089-y)
Supplement: Supplementary file 1 — Supplementary Information [file 42003_2022_4089_MOESM1_ESM.pdf]

Supplementary Data

Supplementary Figures

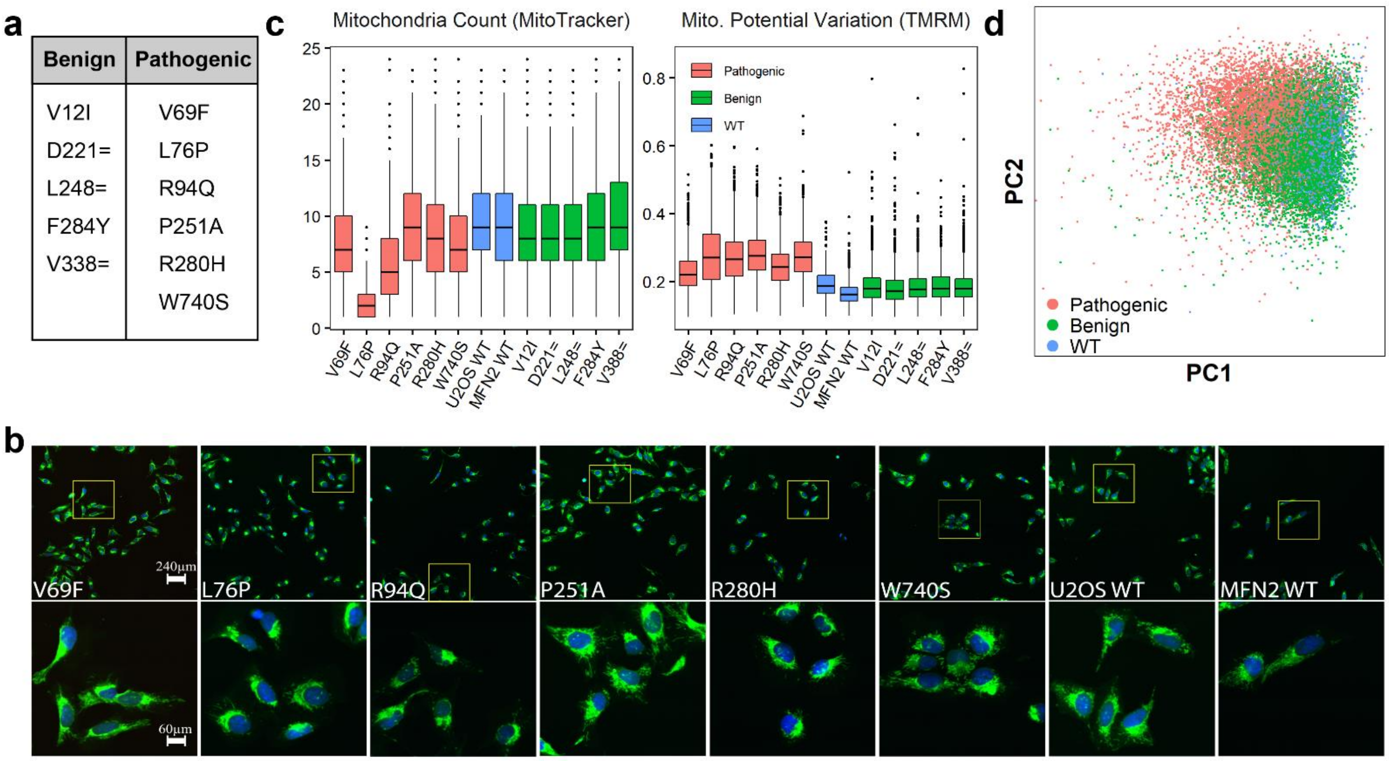

Supplementary Figure 1. Isogenic *MFN2* Mutant Cell Lines Characterization: a. Table of the mutants used. **b.** Confocal images of mutant cells at two magnifications (blue = nuclear staining by Hoechst, green = mitochondria staining by MitoTracker). Scale bars are 240 µm on the top row and 60 µm on the bottom row. **c.** Box plots showing median, range, and interquartile ranges of two features: Mitochondrial count (based on MitoTracker staining) and coefficient of variation in mitochondrial potential (TMRM intensity). Histograms are colored by genotype class (Benign/Pathogenic/WT) (n=18206 total cells, $n_{U2OS\ WT}$=387, $n_{MFN2\ WT}$=1204, n_{V12I}=1793, n_{F184Y}=1058, $n_{D221=}$=1820, $n_{L248=}$=1655, $n_{V388=}$=1657, n_{V69F}=2113, n_{L76P}=1204, n_{R94Q}=2103, n_{P251A}=1195, n_{R280H}=1263, n_{W740S}=754). **d.** Scatter plot of PCA using a set of 26 features measured with TMRM or MitoTracker staining. Each dot represents a single cell, colored by its genotype class (WT/benign/pathogenic) (n=18206 total cells).

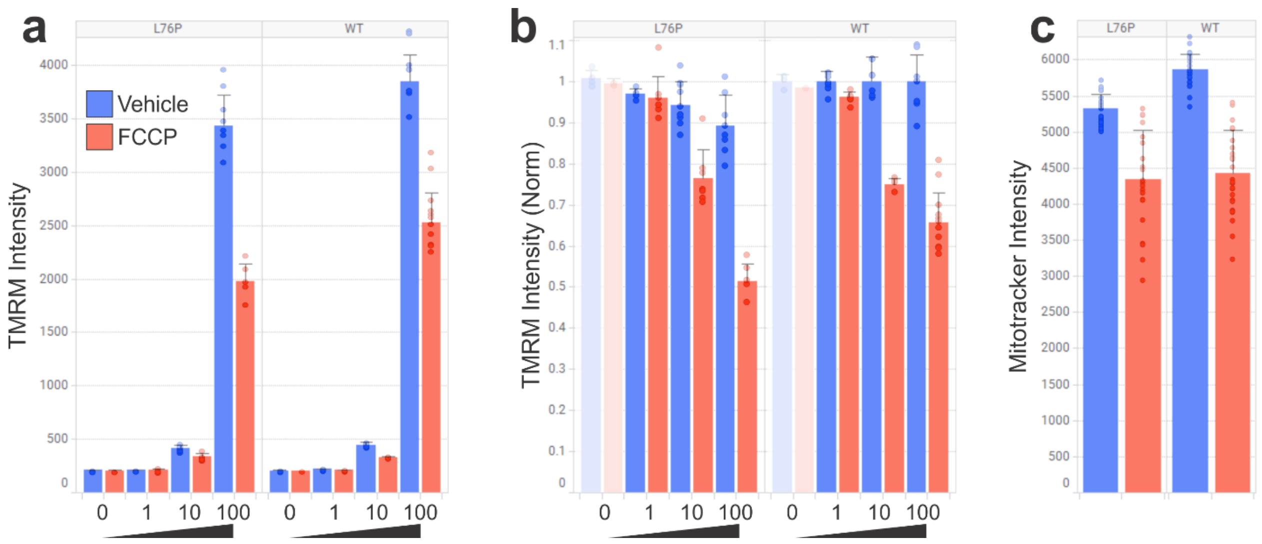

Supplementary Figure 2. Mitochondrial Staining optimization with the Uncoupler FCCP
MFN2 WT and L76P mutant U2OS cells were grown up in T75 vessels. FCCP was added at a concentration of 50µM. Each condition (MFN2 WT FCCP/Non FCCP and L76P FCCP/Non FCCP) was equally and randomly plated across two 96 well plates at a density of 4000 cells/well using a Biomek i5 liquid handler and incubated overnight at 5% CO2, 37C. The following day, the plates were stained with Hoechst, MitoTracker Deep Red, and Tetramethyl Rhodamine methyl ester (TMRM). Concentrations of TMRM were varied equally across both plates: 100nM, 10nM, 1nM, 0nM. **a**. Average TMRM intensity, **b**. average normalized TMRM intensity, **c**. average Mitotracker intensity. Error bars are standard deviation of well replicates **a,b** $n_{0,1}$ = 3 $n_{10,100}$=8, **c** n=30. Normalization was done per TMRM concentration on the WT vehicle treated cells. When FCCP was used, TMRM fluorescence and Mitotracker fluorescence was significantly reduced as compared to the controls, supporting dye specificity to mitochondria, with the 100 nM concentration appearing to give the greatest dynamic range. Among the 4 concentrations of TMRM, there was no significant change in number of cells between all four conditions (not shown).

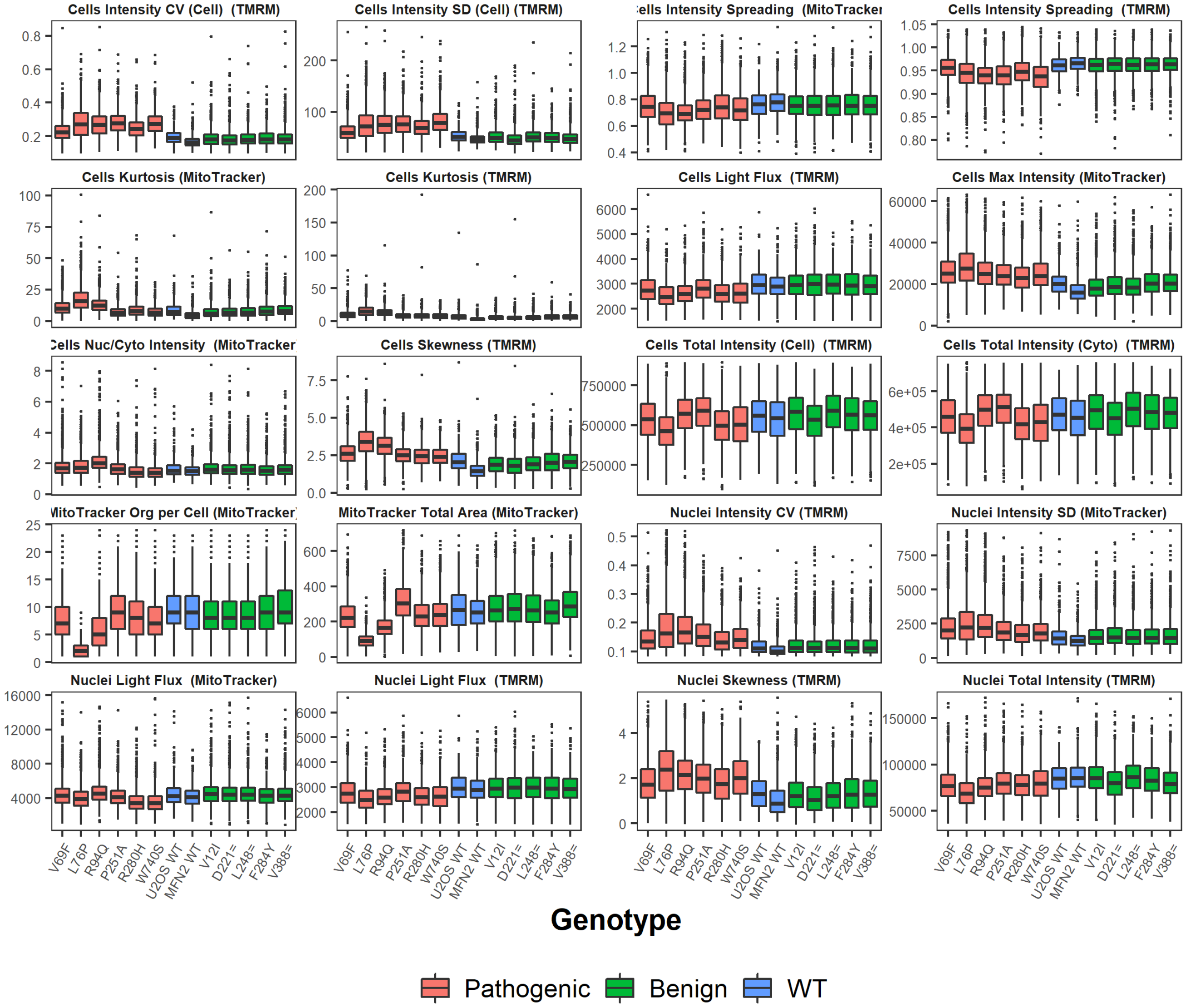

Supplementary Figure 3: Box plots showing showing median, range, and interquartile ranges of important features extracted from TMRM and MitoTracker staining. Boxes are colored by whether the cell was wild type (unmodified wild type or modified with a wild type MFN2 cDNA), contained MFN2 cDNA containing a benign mutant, or contained MFN2 cDNA containing a pathogenic mutant. Features that showed no apparent difference between mutants were omitted (n=18206 total cells, $n_{U2OS\ WT}$=387, $n_{MFN2\ WT}$=1204, n_{V12I}=1793, n_{F184Y}=1058, $n_{D221=}$=1820, $n_{L248=}$=1655, $n_{V388=}$=1657, n_{V69F}=2113, n_{L76P}=1204, n_{R94Q}=2103, n_{P251A}=1195, n_{R280H}=1263, n_{W740S}=754).

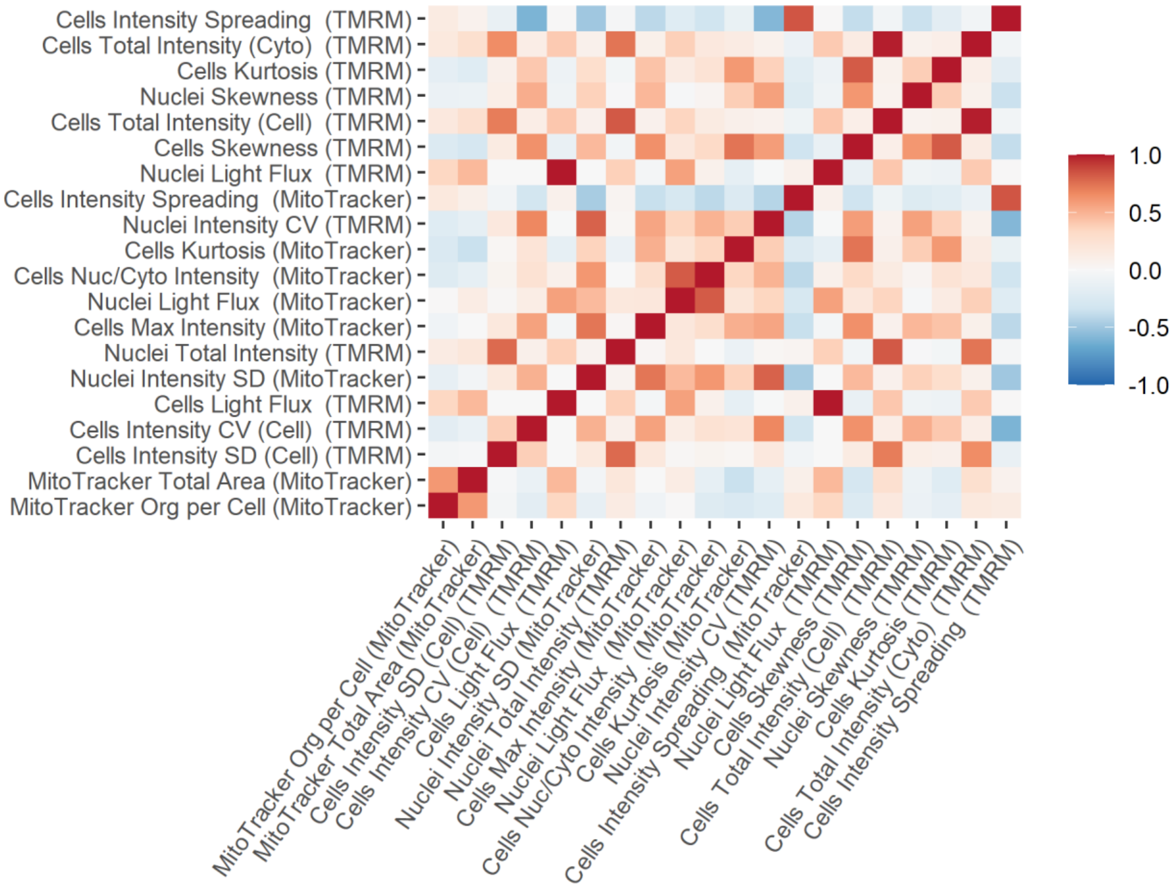

Supplementary Figure 4: Feature correlation plot. Features were chosen based on whether they showed apparent difference between pathogenic mutant and WT cell lines. The final parenthesis indicates the stain that the stated feature is measured on and (Cell)/(Cyto) indicate whether the feature was measured throughout the cell or just the cytoplasm, respectively.

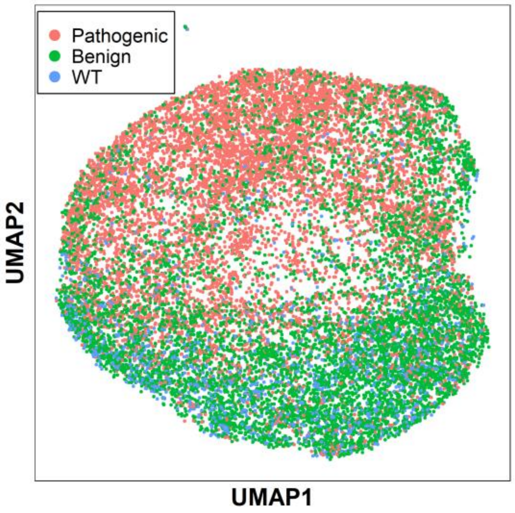

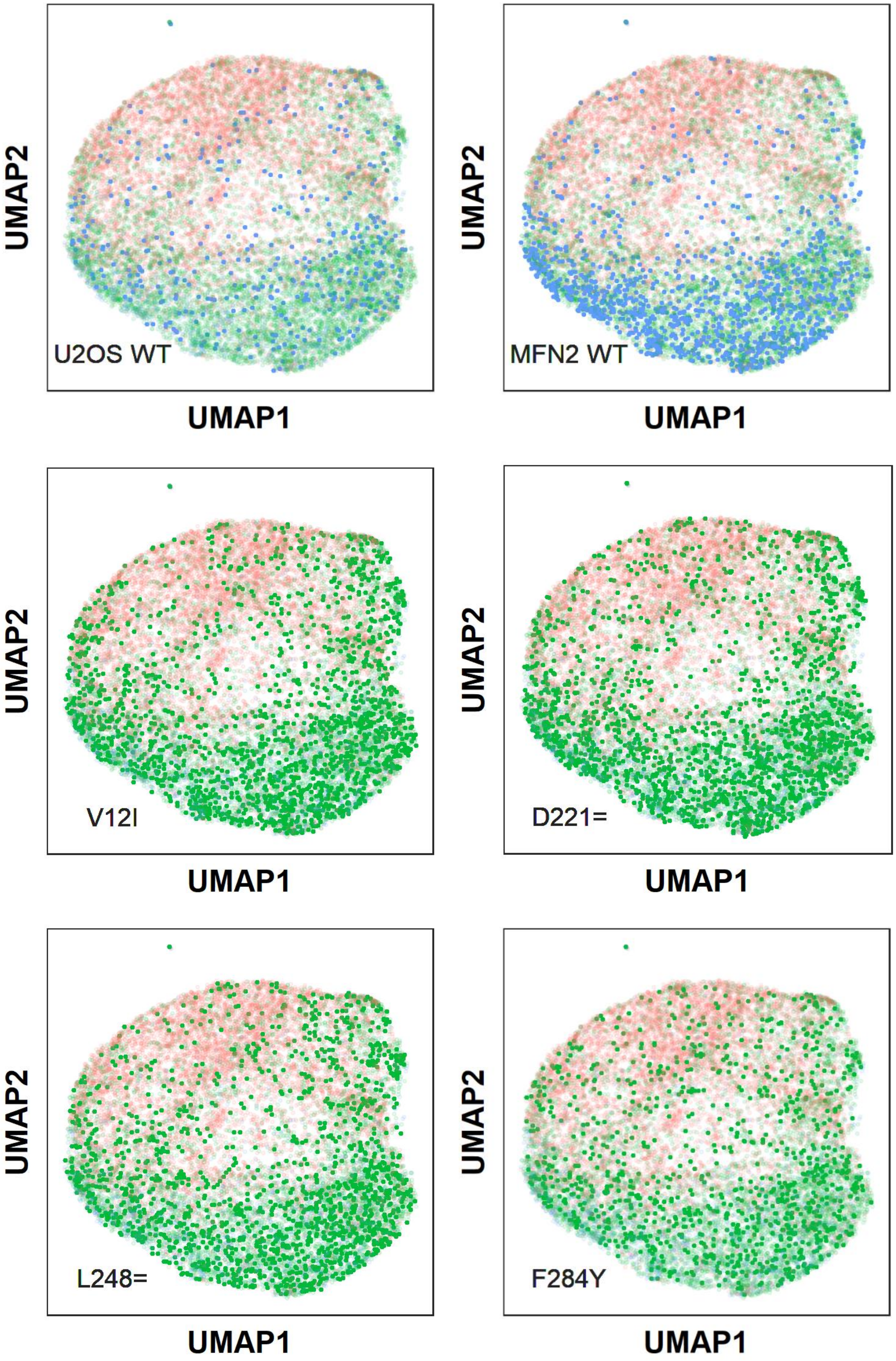
U2OS WT
MFN2 WT
V12I
D221=
L248=
F284Y
UMAP1
UMAP2

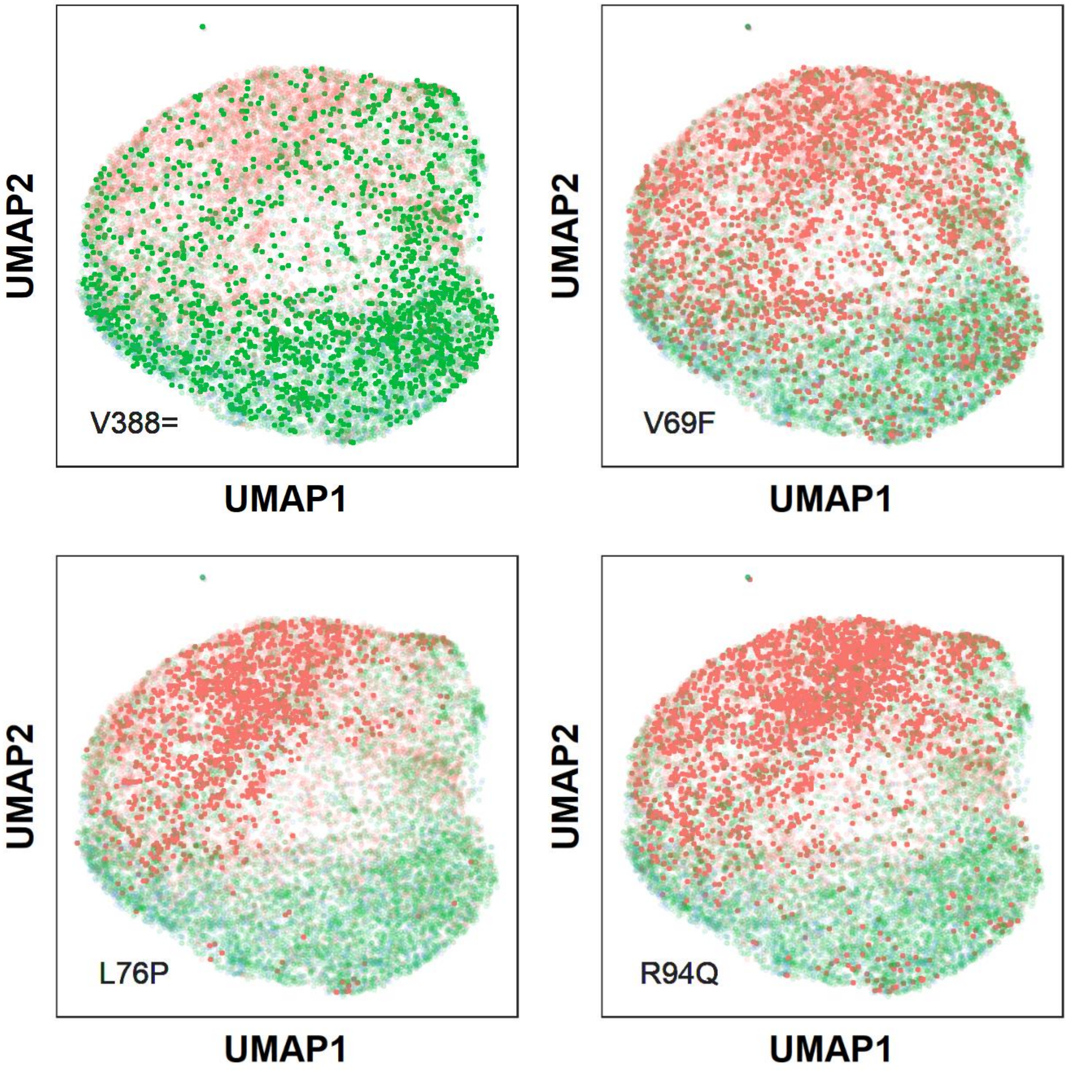

UMAP2
UMAP1
V388=
V69F
L76P
R94Q

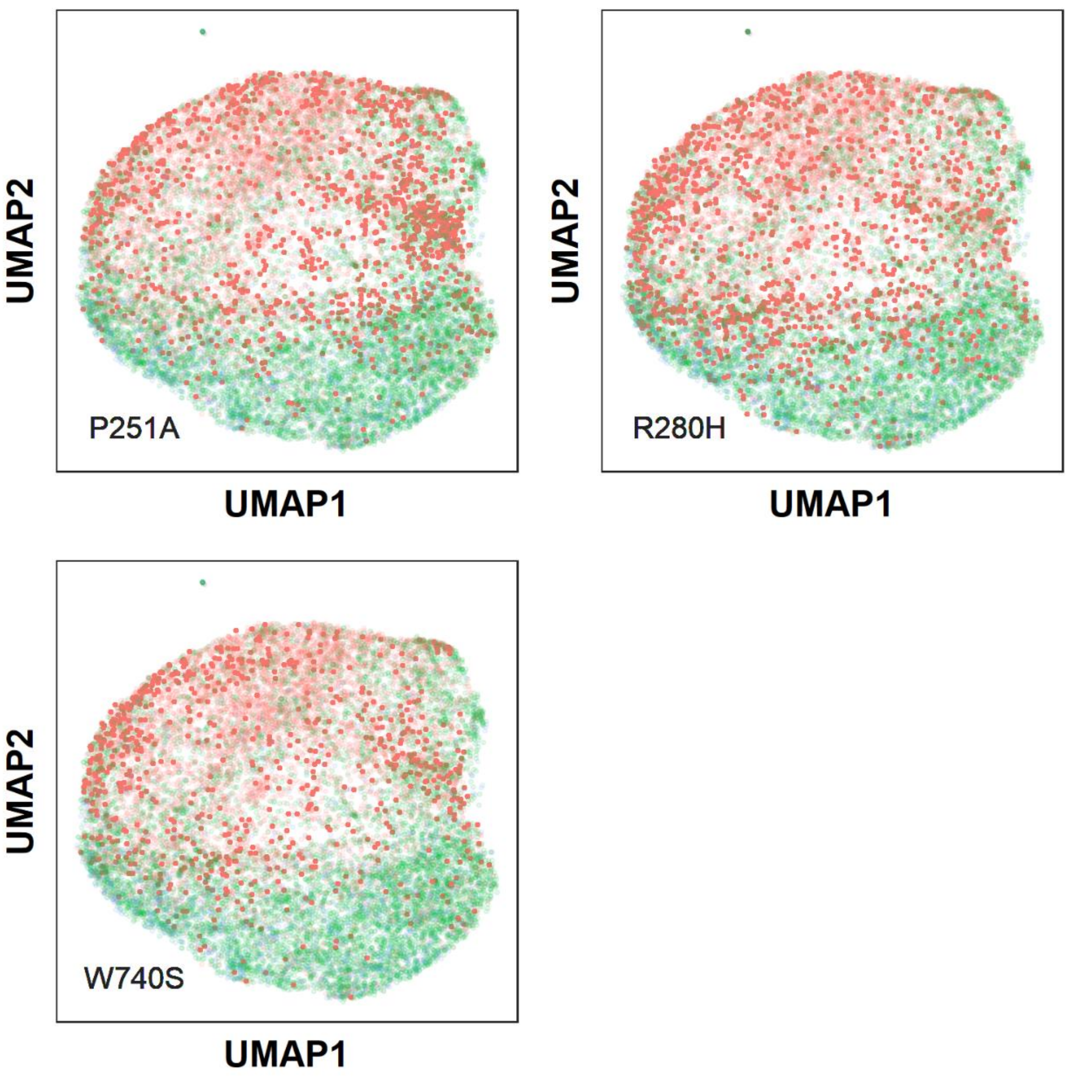

Supplementary Figure 5: Scatter plot for cell feature data UMAPs, highlighted by cell genotype. In *blue*, U2OS WT refers to cells uninfected with lentivirus, while *MFN2* WT refers to cells infected with lentivirus containing the wild type *MFN2* gene. Otherwise, the highlighted genotype refers to the mutation present in the introduced *MFN2* gene (*red* pathogenic, *green* benign) (n=18206 total cells $n_{U2OS\ WT}$=387, $n_{MFN2\ WT}$=1204, n_{V12I}=1793, n_{F184Y}=1058, $n_{D221=}$=1820, $n_{L248=}$=1655, $n_{V388=}$=1657, n_{V69F}=2113, n_{L76P}=1204, n_{R94Q}=2103, n_{P251A}=1195, n_{R280H}=1263, n_{W740S}=754).

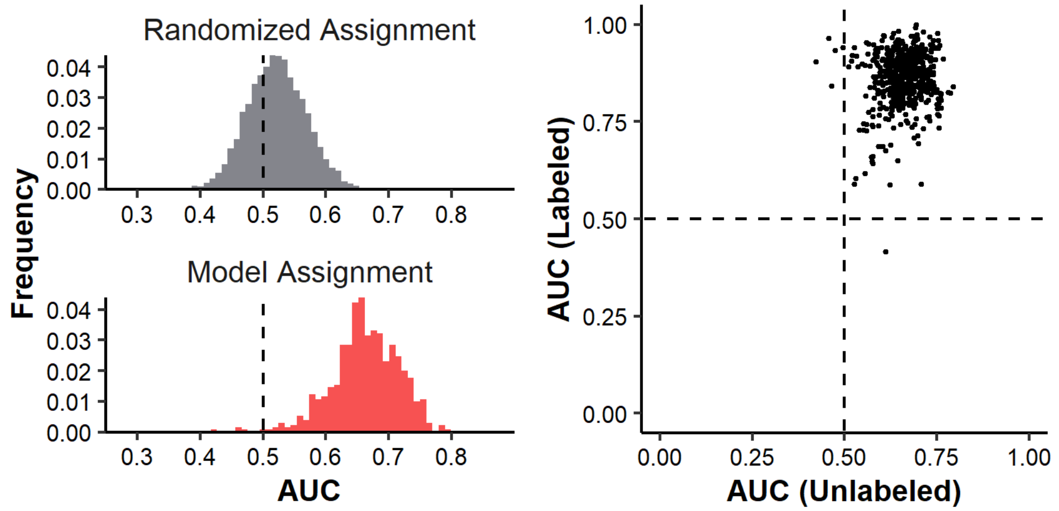

Supplementary Figure 6 Model AUCs for identifying a mixture of wild type and six pathogenic mutant cell lines. Left: A histogram of AUCs for models detecting mutants in the unlabeled population of wild-type cells and mutants. On top, a histogram of AUCs generated from randomly assigning models is shown as a comparison. **Right**: A scatterplot showing the performance of 610 models in detecting mutants in a mixture of wild type cells and four pathogenic mutants. Each point represents a model and its position is determined by its ability to distinguish cells in the pure control wells and cells in the mixed wells.

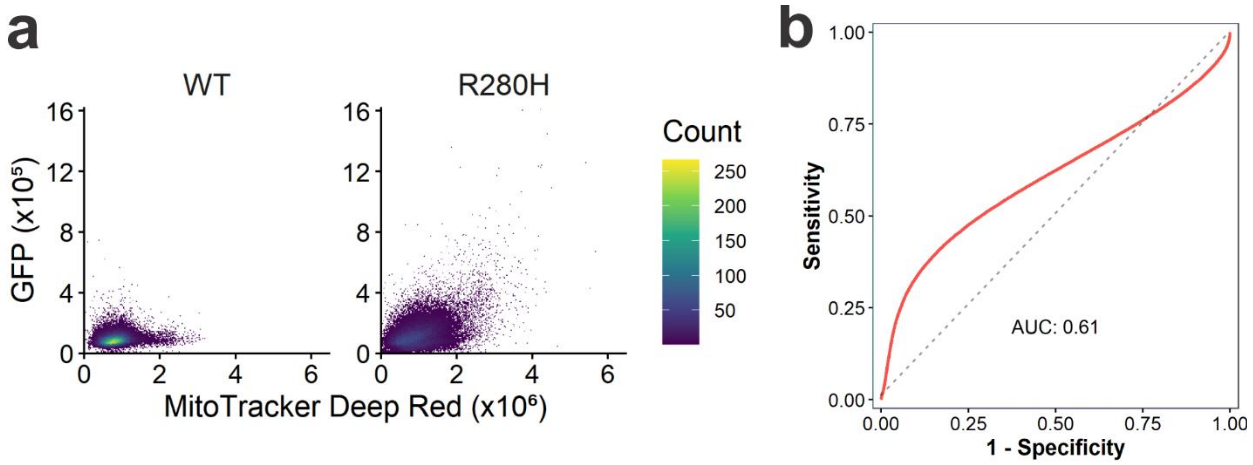

Supplementary Figure 7 Modeled ROC from flow cytometry. a. Side-by-side flow cytometry scatter plots of the *MFN2* WT and R280H mutant cell (GFP was present equally in both cell lines). **b**. ROC Curve based on flow data for *MFN2* WT vs. *MFN2* R280H mutants on the PerCP-Cy5.5-A-Compensated channel.

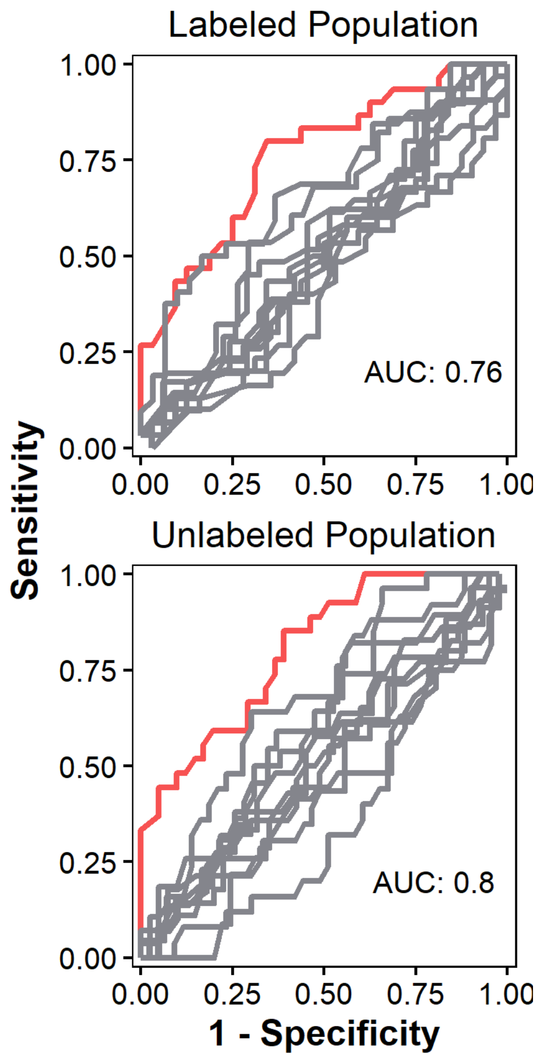

Supplementary Figure 8 Results from Raft-Seq between P251A Pathogenic and D221= Benign. ROC Curves which were generated using the best model, separated by data that was generated from cells picked from wells in the labeled cell population (upper) and from the unlabeled cell population (lower). The red curves are for the models in question and the grey curves are for control models generated by random shuffling of labels.

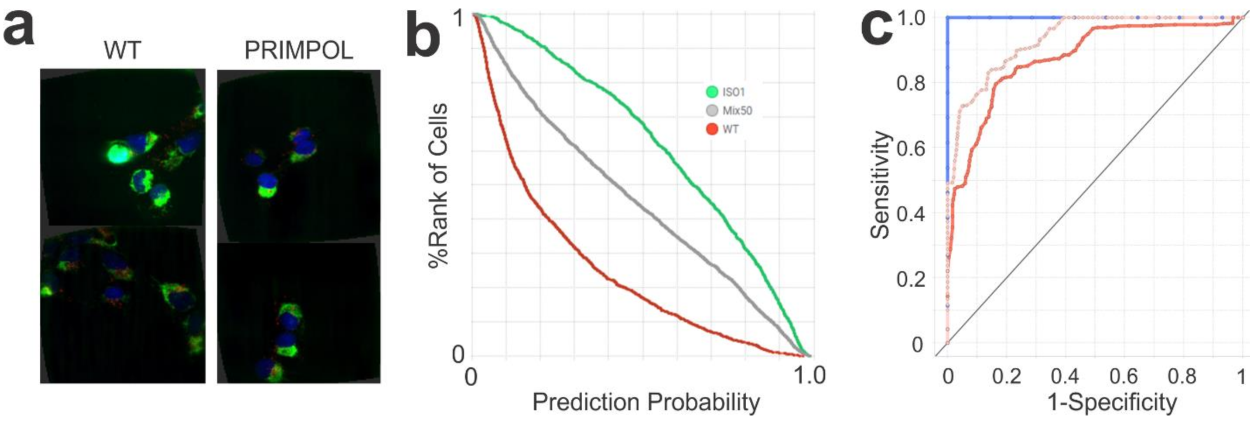

Supplementary Figure 9 Raft-seq can detect mitochondrial dynamic alterations induced by endogenous mutations in the mitochondrial primase PRIMPOL. a. Confocal imaging of wild-type and perturbed cells. U2OS cells were stained with Hoechst and Mitotracker. Each image is 200 µm wide. **b.** Ranked histogram comparing model prediction scores of a population of wild-type cells (red, WT), PRIMPOL perturbed cells (green, ISO1), and an admixture of the two (gray, Mix50). Receiver operating curves from wells containing a mixture of WT and ISO1 cells (AUC: 0.8744 Actual red, 0.9299 Predicted pink). Modeled with Boosted Tree algorithm using the following features: (Hoechst): Nuclei Diameter, Perim, Area, Displacement. Intensity, Max Inten, Inten SD, CV, Texture Energy, Entropy. (Mitotracker): Mito Area, Total Area, Distance to Nuc, Neighbor Count, Spacing, Form Factor. Intensity, Max, SD, CV, Total, Spreading, Flux (for nuclei, cytoplasm, whole cell, mito), Texture Energy, Entropy, Kurtosis, Nuc/Cyto Intensity.

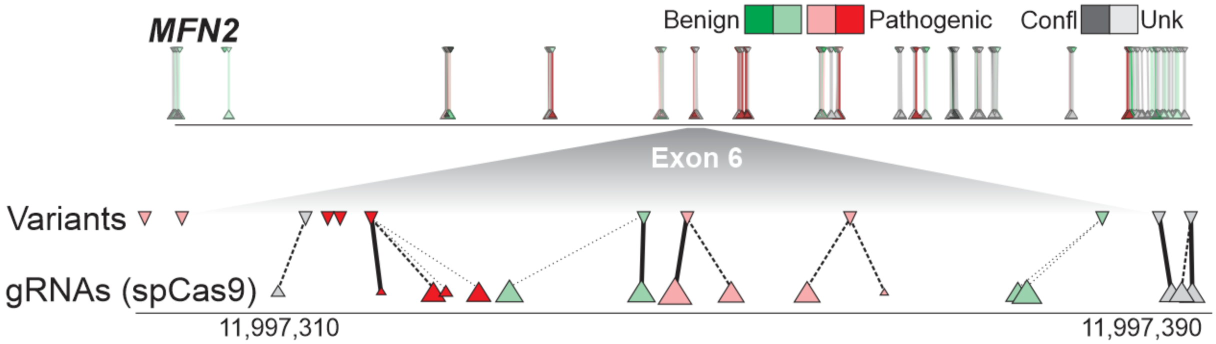

Supplementary Figure 10. Diagram of coding sequence with ClinVar variants (downward facing triangles) and their closest gRNA cut sites (upward facing triangles). The upper line shows the entire genomic context of the MFN2 gene, while the lower inset magnifies the variants and gRNA cut sites for exon 6. Lines indicate the ‘partner’ gRNAs for each ClinVar mutation, where thicker lines indicate closest association.

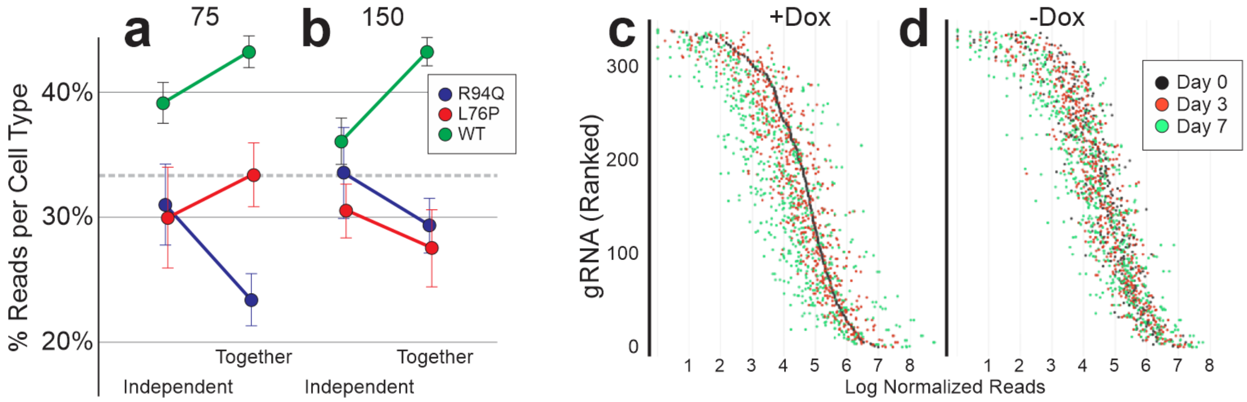

Supplementary Figure 11. Fitness consequences of MFN2 mutations. a,b. U2OS stable lines containing R94Q, L76P, mutations, or the WT control line were grown in separate wells or evenly mixed together. This was done at 2 densities 75,000 (**a**) and 150,000 (**b**) cells / well of a 6-well plate and cells were allowed to grow for 4 days. Cells grown independently (n=3) were harvested into the same tube (so all subsequent steps would be together). All cells were taken through the genotyping pipeline using the same primers as above, then the reads were counted, and the proportion of each cell type measured. **c,d**. A similar experiment was performed with the scanning MFN2-targeted gRNAs, under Dox (**c**) to induce Cas9 cutting, or under control conditions (**d**) with the gRNAs, but no Dox (and therefore no Cas9 cutting). Samples were taken initially, then at 3 and 7 days after Dox induction. Bulk timepoint samples were processed through the genotyping pipeline and reads were counted for each gRNA. The open-source software package DrugZ was used to analyze the results to look for gRNAs which significantly altered survival over this time course. The analysis showed that there was no significant changes in representation between the cells that had been exposed to doxycycline and the cells that had not, and thus no significant change in cell viability between the two conditions, even though mitochondrial differences were observed between the two groups.

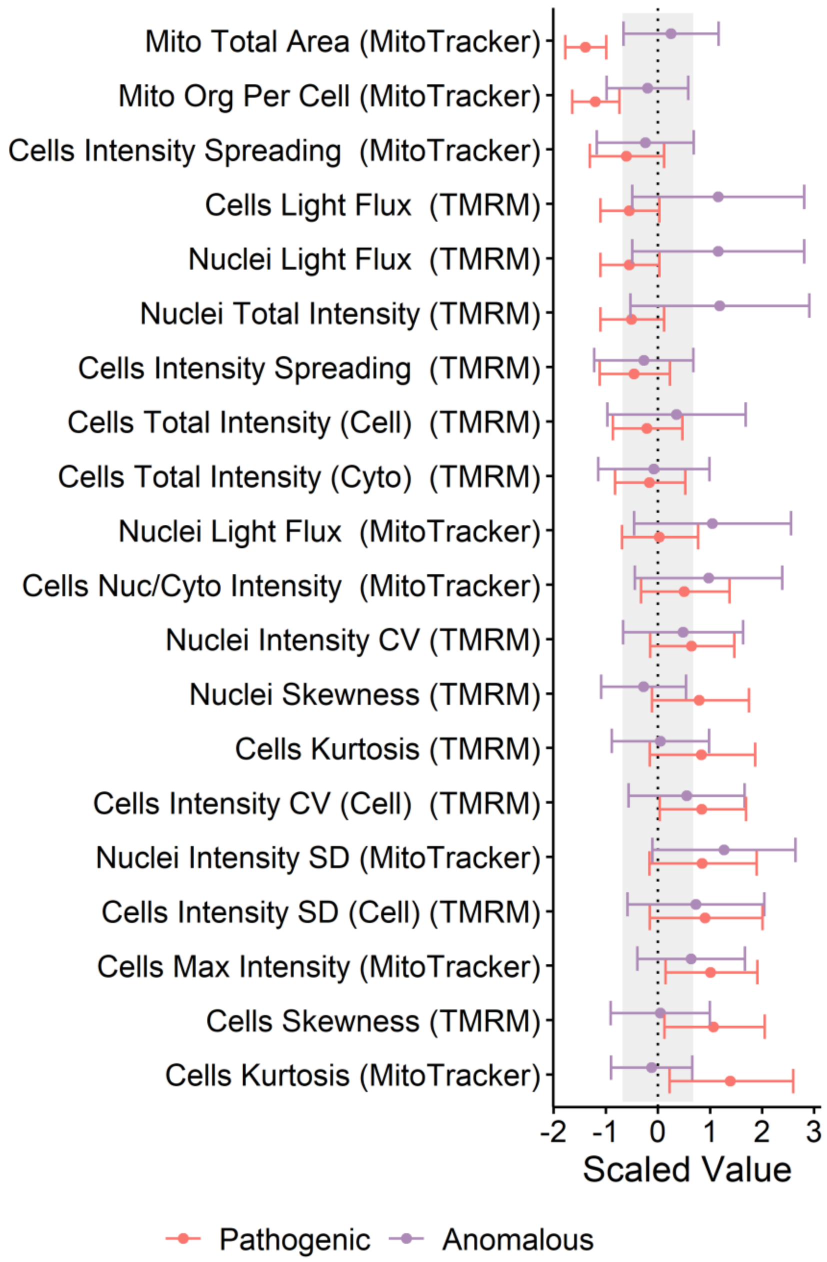

Supplementary Figure 12. Isogenic Feature Comparison. We compared the distributions of the important features identified earlier for anomalous cells and strong pathogenic mutants (L76P & R94Q) by normalizing each to the distribution of the non-anomalous and benign mutants, respectively. Some of the features covaried between pathogenic and anomalous, and this pattern was stronger than in the non-anomalous (pathogenic vs. non-anomalous r^2 -0.02, vs anomalous r^2 0.001, neither significant). Red is for the distribution in the pathogenic cells; purple is for the anomalous cells. Background grey shading indicates the base distribution for the non-anomalous/benign cells. Bar width and ranges correspond to 0.687σ in both directions, covering 50% of the data. Anomalous cells are the top 0.5% highest anomaly-detection scores from 152 models.

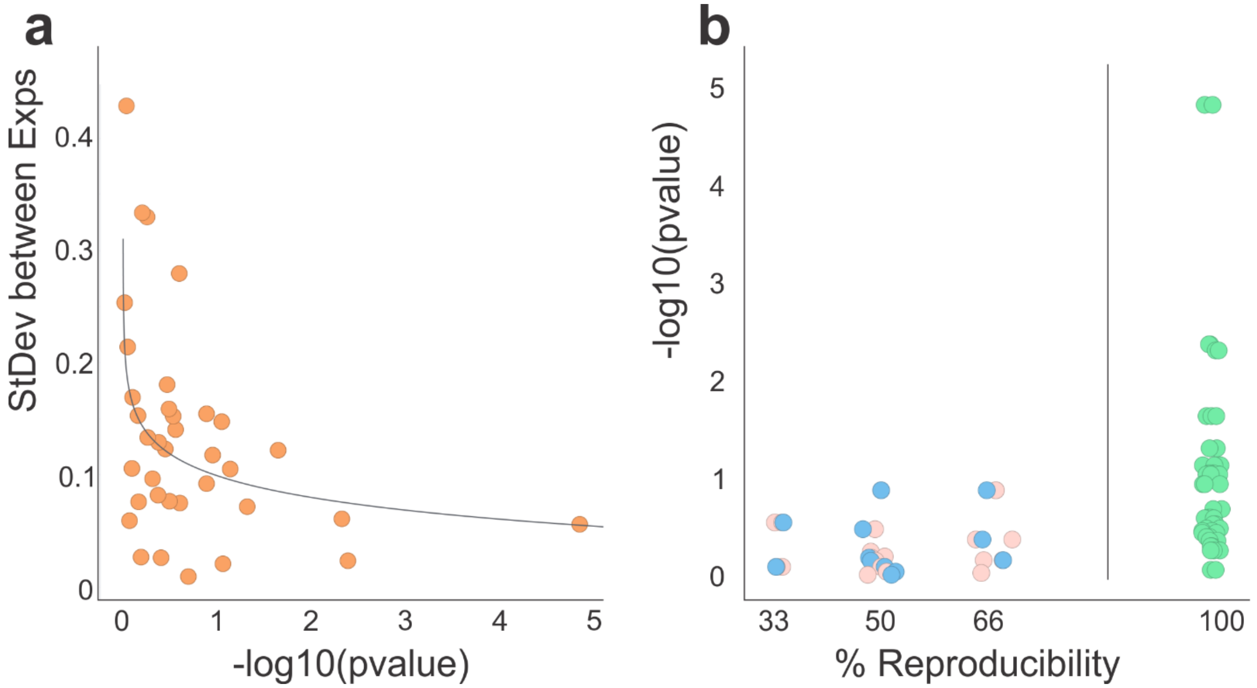

Supplementary Figure 13. Raft-Seq Screening Reproducibility. Of the gRNAs that were represented in at least 2 replicate experiments and had at least 2 observations in each experiment (79 in total), we examined the relationship between the gRNA's p-value in the screen and factors that measure reproducibility. **a.** We examined the standard deviation between the mean prediction probabilities for each experiment, divided by the average prediction probability for the gRNA across all observations in all experiments (lower is more reproducible). The curve fit indicates the expected result and highlights that hits with significant p-values are very reproducible, but it can also be seen that gRNAs with low p-values often had low standard deviations, indicating that they were consistently near the middle of the distribution. **b.** We directly measured %reproducibility by asking what % of replicate experiments were consistently above or below the 0.5 ensemble score cutoff. 59.6% of gRNAs are colored in green and were 100% reproducible between experimental replicates. Of the remaining gRNAs, we asked what % of experiments were within 0.16 of that gRNA's global average and found an additional 15% (colored blue), bringing the total reproducible gRNAs to 74.7%. Therefore, with phenotypes of similar penetrance and expressivity, and with models that are of similar predictive power, we would expect a reproducibility of around 75%.

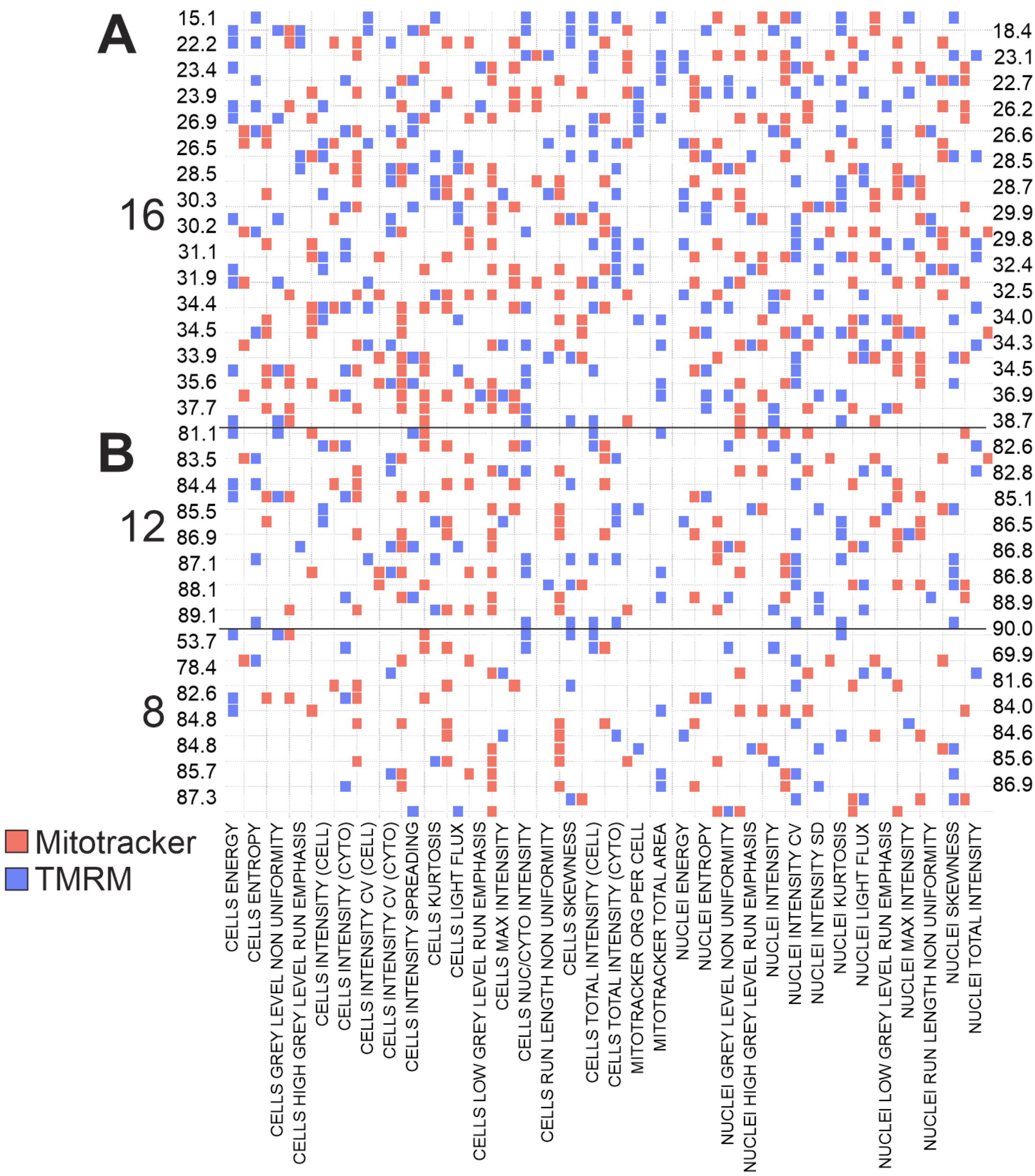

Supplementary Figure 14: MFN2 Isogenic Lines carrying mutations are distinct from WT. Decision Jungles were trained on either WT vs. Known KO clones (**A**) or known mutants and known un-cut clones (**B**) with either 16, 12, or 8 features per model. Each model had a random set of features it could use in training. Then, the model was deployed and the Kruskall-Wallis p-Value comparing the model scores of the UTR/Coding mutants with the WT clones was assessed (these p-values are displayed as -log10 on either side of the graph).

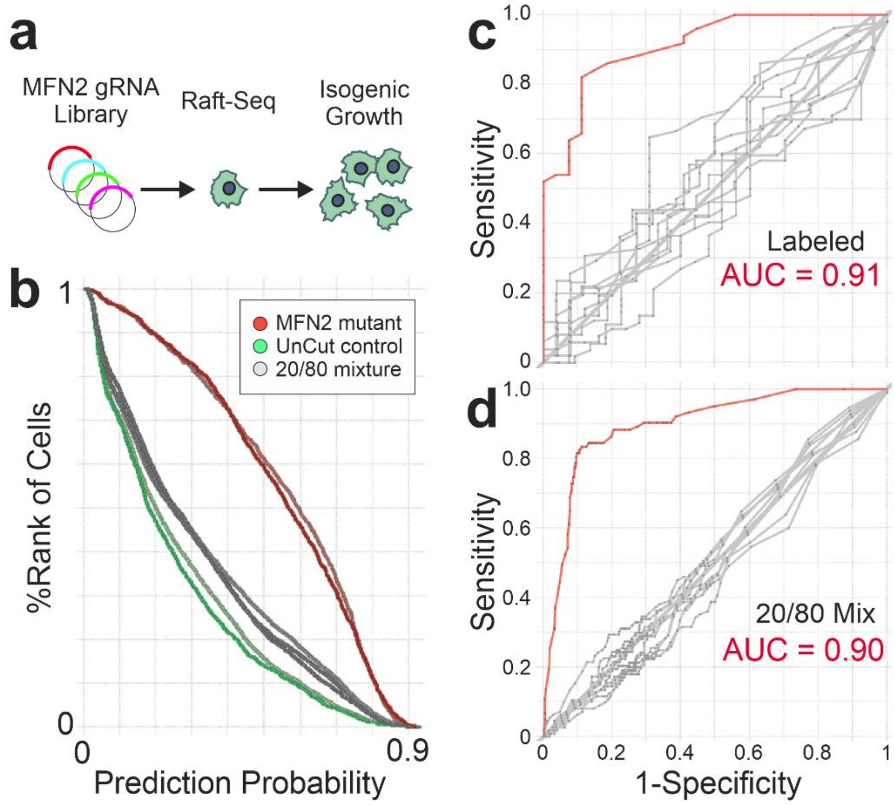

Supplementary Figure 15. Endogenous MFN2 Mutant Line Can Be Effectively Separated from a Non-Cutting Control. a. U2OS cells with were screened against the MFN2 gRNA library using Raft-Seq, and single cells were picked for isogenic growth. One line, 5' UTR Ins A04, was used here in a Raft-Seq experiment to compare its ability to separate against an un-cut control. The model was trained in AzureMLStudio (two class decision jungle) with 5 features (Nuclei Area, Mitotracker Intensity Kurtosis, Skewness, Energy and TMRM Intensity Entropy). **b**. Ranked histogram comparing single cells from labeled wells (where only the mutant or control were plated) against their prediction probabilities from the 5-feature model. Cells from the control (labeled) wells, and mixture well (20% mutant, 80% control) were picked and genotyped. **c**. ROC curves for the labeled (control) well (n=77). **d**. ROC curve from the wells with a mixture of the two cell types. The model performed with an AUC = 0.9, accuracy of 88%, precision 69% (n = 490 cells). Red curves are the real data while the gray curves were generated from sets of data where the link between the true genotype and the prediction probability were scrambled (noise models).

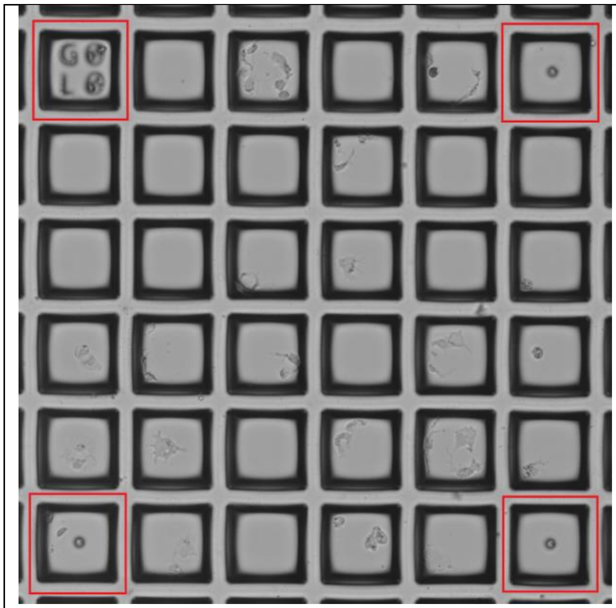

Supplementary Figure 16. Cropped example field of CytoSort raft plate: Alphanumeric fiduciary markers are etched on every 10 rafts (e.g. G0L0), and dot fiduciary markers are etched on every 5 rafts. Rafts with fiduciary markers are highlighted with red outlines.

Supplementary Tables

Feature	Stain
Mito Total Area	Mitotracker Red
Nuclei Low Grey Level Run Emphasis	Mitotracker Red
Mito Intensity	Mitotracker Red
Cells Entropy	Mitotracker Red
Nuclei Intensity CV	Mitotracker Red
Cells Skewness	Mitotracker Red
Cells Total Intensity (Cyto)	Mitotracker Red
Cells Intensity Spreading	Mitotracker Red

Supplementary Table 1: Table of the 8 features used for cell isolation and random feature assembly with the corresponding stain.

Clone Name	Mutation	Type	DNA Cut	gRNA Sequence	Ex	I/I+C
H20fs/WT	H20 Frameshift / WT	Coding	g.chr1:11989226_11989232delCACATGG	AAATAAGAGACACATGGCTG	2	0.376
V459fs/WT	V459 Frameshift / WT	Coding	g.chr1:12004594_12004595insC	CCCTTCTCCAGTAGTCCTCA	12	0.46
P588fs/PQG590del/WT	P588fs / PQG590del / WT	Coding	g.chr1:12006581_12006608delCCCCACTGCCACAGGGCTCGCTCACCCA,<br>g.chr1:12006588_12006596delGCCACAGGG	ACTGCCACAGGGCTCGCTCA/C<br>ACTGCCACAGGGCTCGCTC	16	0.31
LEH710del/WT	LEH710del / 1+ WT	Coding	g.chr1:12009648_12009656delACCTGGAGC	CACCCGGGAGAACCTGGAGC	18	0.24
VS507del/PV506del	Exon 15, VS507del/PV506del	Coding	g.chr1:12005732_12005737delCTGTGT,<br>g.chr1:12005731_12005736delCCTGTG	GTGTCTGTGCGGAGTCAGAT	15	0
SRT612del/S615del/S615fs	Exon 16, SRT612del / S615del / S615fs	Coding	g.chr1:12006655_12006664delTCCAGGACC,<br>g.chr1:12006662_12006665delCCT,<br>g.chr1:12006662_12006672delCCTCCATGGG	GGACCTCCATGGGCATTCTT	16	0
5'UTRdel A2P2-B05	5' UTR Deletion (3 var)	UTR	g.chr1:11980300_11980309delGAGGCGTAAG,<br>g.chr1:11980302_11980332delGGCGTAAGGAGTAGGCGGGGCGAGCCGGCTG,<br>g.chr1:11980312_11980340delGACTCGGGTCGGCCGAGCGGGGCGGATGA*	GCAGAGGCGTAAGGAGTAGG	1	0
5'UTRdel/WT A3P1-E02	5' UTR Deletion / 2+ WT	UTR	g.chr1:11980421_11980431delCTGGGGTGGCG	CTGGGGTGGCGCTCGCTGGT	1	0.38
5'UTRins A1P2-A04	5' UTR Insertion (3 var)	UTR	g.chr1:11980347_11980348insAAG,<br>g.chr1:11980348_11980349insCCC,<br>g.chr1:11980349_11980350insGGTC	CCAGCTCACCCGGGTCGAGG	1	0
5'UTRdel/WT A1P1-F08	5' UTR Deletion / 2 WT	UTR	g.chr1:11980421_11980431delCTGGGGTGGCG	CCTGGGGTGGCGCTCGCTGG	1	0.35
5'UTRdel/WT A2P1-D02	5' UTR Deletion / 2 WT	UTR	g.chr1:11980421_11980431delCTGGGGTGGCG	CCTGGGGTGGCGCTCGCTGG	1	0.34
5'UTRdel/WT A3P2-A05	5' UTR Deletion / 2 WT	UTR	g.chr1:11980421_11980431delCTGGGGTGGCG	CCTGGGGTGGCGCTCGCTGG	1	0.36
5'UTRdel A3P3-C09	5' UTR Deletion, No WT	UTR	g.chr1:11980286_11980288delGTC	AGTCGCGGGGCAGCAGAGGC	1	0
5'UTRdel A3P3-D05	5' UTR Deletion, No WT	UTR	g.chr1:11980414_11980419delCCTCCC	CCCCCTGGGGTGGCGCTCGC	1	0
3'UTRdel A2P3-E04	3' UTR Deletion	UTR	g.chr1:12012466_12012472delACACAGG,<br>g.chr1:12012468_12012472delACAGG,<br>g.chr1:12012689T>C	CACAGGACAGCTGGAGAATG	19	0
3'UTRdel/WT A2P1-A08	3' UTR Deletion (2 different) / 1 WT	UTR	g.chr1:12013130_12013170delCCACTTCACAGCATGTCAGGGAAAATCACTGTCACACAATT, g.chr1:12013195delA	GGCCACTTCACAGCATGTCA/G<br>GGCCACTTCACAGCATGTC	19	0.09
3'UTRins A2P1-C06	3' UTR Indel (2 different, No WT)	UTR	g.chr1:12013045_12013046insT,<br>g.chr1:12013195delA	TCAGTCTGTCCTGTTGTGTG	19	0
3'UTRdel/WT A2P2-C04	3' UTR Deletion / 1 WT	UTR	g.chr1:12013094_12013104delTGTTCCCGGCG	ATGCTGTGGGTGGATGTTCC	19	0.23
3'UTRdel A2P2-E05	3' UTR Deletion (2 different) No WT	UTR	g.chr1:12012811_12012828delGTGAGAAAAGCAGTTTGG,	CTGTGTGAGAAAAGCAGTTT	19	0

			g.chr1:12012815_12012830delGAAAAGCAG TTTGGGT			
3'UTRdel/WT A2P3-A04	3' UTR Deletion / WT	UTR	g.chr1:12012692_12012724delCCACCCTCC CTGATCTCCAGAACCTTCGACTGA	TCGACTGACCCCCTTGTCTT	19	0.26
3'UTRdel/WT A1P1-C06	Exon 19, 3' UTR Deletion (large)	UTR	g.chr1:12012447 [large del]	TCCACTTGGCCTCGTTCTGC	19	0.32
WT A3P2-F10	WT	WT		CCTGGGGTGGCGCTCGCTGG		0.5
WT A2P3-G09	WT	WT		CCTGGGGTGGCGCTCGCTGG		0.48
WT A2P2-F12	WT	WT		CCTGGGGTGGCGCTCGCTGG		0.46
WT A3P3-C08	WT	WT		CTGGGGTGGCGCTCGCTGGT		0.51
WT A2P2-F05	WT	WT		AAGGTGGGGACGTTGGTGGC		0.52
WT A2P3-G12	WT	WT		AGAAGAAGATCAATGGCATT		0.49
WT A3P3-D08	WT	WT		ATTGCTTCCTGCGGGTAGAG		0.52
WT 3E5	WT	WT	WT Parental Clone	N/A		0.5

Supplementary Table 2: *MFN2* Isogenic mutant line information table. The U2OS isogenic lines were genotyped to determine the gRNA and the editing consequence of Cas9 cutting. The table shows the 'Clone Name' used in other parts of the paper as well as the longer mutation definition for the clone and the specific genomic mutation. Also listed is the sequence of the gRNA used, the exon where the cut was located, and the % of intact target reads as a ratio with a control (constant) sequence, where 0 = all alleles are mutant, and 0.5 = all alleles are WT. Variant calling was performed after paired end reads were merged using the fastq-join method from ea-tools (https://github.com/ExpressionAnalysis/ea-utils). Merged fastq files were then aligned using BWA-mem (DOI:10.1093/bioinformatics/btp324). Annotated variant call files were produced using Picard Tools (http://broadinstitute.github.io/picard/) and the Genome Analysis Tool Kit (DOI: 10.1101/201178).

Mitochondrial Feature	KW p-value
CELLS ENTROPY WVT	2.72E-17
CELLS ENTROPY WVM	4.40E-16
CELLS KURTOSIS WVT	2.62E-15
CELLS ENERGY WVT	8.06E-15
CELLS KURTOSIS WVM	2.06E-13
CELLS SKEWNESS WVM	3.11E-13
CELLS INTENSITY (CYTO) WVM	3.10E-12
CELLS ENERGY WVM	1.59E-10
CELLS INTENSITY (CELL) WVM	1.64E-10
CELLS INTENSITY (CYTO) WVT	2.42E-10
CELLS GREY LEVEL NON UNIFORMITY WVM	2.60E-10
CELLS RUN LENGTH NON UNIFORMITY WVT	3.41E-10
NUCLEI ENTROPY WVT	4.33E-10
CELLS RUN LENGTH NON UNIFORMITY WVM	4.78E-10
CELLS LOW GREY LEVEL RUN EMPHASIS WVT	7.24E-10
CELLS INTENSITY (CELL) WVT	2.49E-09
CELLS SKEWNESS WVT	6.26E-09
NUCLEI ENERGY WVT	9.21E-09
CELLS TOTAL INTENSITY (CYTO) WVT	2.23E-08
CELLS TOTAL INTENSITY (CELL) WVT	1.21E-07
NUCLEI ENTROPY WVM	2.66E-07
CELLS TOTAL INTENSITY (CYTO) WVM	9.80E-07
CELLS TOTAL INTENSITY (CELL) WVM	3.76E-06
NUCLEI LOW GREY LEVEL RUN EMPHASIS WVT	7.18E-06
CELLS GREY LEVEL NON UNIFORMITY WVT	8.21E-06
NUCLEI RUN LENGTH NON UNIFORMITY WVT	2.34E-05
CELLS MAX INTENSITY WVT	3.08E-05
NUCLEI GREY LEVEL NON UNIFORMITY WVM	5.76E-05
NUCLEI TOTAL INTENSITY WVT	1.00E-04
NUCLEI SKEWNESS WVM	2.04E-04
CELLS LOW GREY LEVEL RUN EMPHASIS WVM	4.76E-04
NUCLEI INTENSITY WVT	5.42E-04
NUCLEI TOTAL INTENSITY WVM	5.72E-04
NUCLEI MAX INTENSITY WVT	5.93E-04
NUCLEI INTENSITY WVM	8.27E-04

Supplementary Table 3: Individual mitochondrial features that distinguish the isogenic mutants from un-cut clones. The name of the engineered feature and the Kruskal-Wallis p-Value distinguishing the single feature’s mean difference between the cut isogenic clones (UTR or coding), and the WT isogenic clones. WVM refers to Mitotracker and WVT refers to TMRM. Features with Level in the name are texture-based features.

Clone	Baseline	FCCP	Oligomycin	ROT/AA
iCas9-3E5	1 (0.99/1)	2.02 (2/2.04)	0.39 (0.38/0.39)	0.27 (0.27/0.27)
WT A2P2-F12	1.02 (1.01/1.03)	1.5 (1.43/1.58)	0.44 (0.42/0.46)	0.25 (0.24/0.25)
WT A2P3-G12	0.8 (0.79/0.81)	1.69 (1.66/1.72)	0.34 (0.34/0.34)	0.26 (0.26/0.27)
WT A3P3-C08	0.77 (0.77/0.78)	1.52 (1.46/1.58)	0.27 (0.27/0.27)	0.21 (0.21/0.21)
WT A3P3-D08	1.02 (1.01/1.02)	1.86 (1.78/1.95)	0.42 (0.41/0.43)	0.29 (0.29/0.3)
3'UTRdel/WT A2P3-A04	0.97 (0.95/0.98)	2.02 (2/2.04)	0.32 (0.32/0.32)	0.21 (0.21/0.22)
5'UTRdel A2P2-B05	0.95 (0.93/0.97)	2.11 (1.97/2.25)	0.33 (0.33/0.33)	0.22 (0.21/0.22)
5'UTRins A1P2	0.88 (0.88/0.89)	1.77 (1.74/1.8)	0.34 (0.34/0.34)	0.25 (0.25/0.25)
3'UTRdel A2P3-E04	0.87 (0.86/0.88)	1.99 (1.98/2.01)	0.34 (0.34/0.35)	0.2 (0.2/0.21)
3'UTRdel/WT A2P1-A08	0.74 (0.73/0.76)	1.48 (1.45/1.5)	0.29 (0.28/0.29)	0.19 (0.19/0.2)
3'UTRdel A2P2-E05	0.72 (0.71/0.72)	1.6 (1.58/1.62)	0.26 (0.25/0.26)	0.19 (0.18/0.19)
MFN2 L76P	0.7 (0.7/0.71)	1.2 (1.18/1.21)	0.28 (0.28/0.28)	0.2 (0.2/0.2)
H20fs/WT	0.68 (0.67/0.68)	1.29 (1.28/1.31)	0.28 (0.28/0.28)	0.21 (0.21/0.21)
V459fs/WT	0.6 (0.59/0.61)	1.05 (1.01/1.09)	0.22 (0.21/0.22)	0.15 (0.15/0.15)
VS507del/PV506del	0.56 (0.55/0.58)	1 (0.97/1.03)	0.23 (0.23/0.24)	0.17 (0.17/0.17)
3'UTRdel/WT A2P2-C04	0.55 (0.54/0.56)	1.07 (1.05/1.08)	0.18 (0.18/0.18)	0.11 (0.11/0.11)
5'UTRdel A3P3-C09	0.44 (0.43/0.45)	0.77 (0.73/0.8)	0.15 (0.15/0.16)	0.09 (0.09/0.1)
SRT612del/S615del/S615fs	0.31 (0.31/0.32)	0.53 (0.51/0.54)	0.14 (0.14/0.14)	0.1 (0.1/0.1)
3'UTRdel/WT A1P1-C06	0.2 (0.2/0.2)	0.27 (0.26/0.28)	0.09 (0.09/0.09)	0.06 (0.05/0.06)

Supplementary Table 4: Metabolic testing results on the U2OS *MFN2* mutant isogenic cell lines. The ‘Clone Name’ for each isogenic line is given, the average and upper/lower 95% confidence intervals (in parentheses) of the OCR (pMoles/min) normalized to parental WT control (iCas9-3E5). The 4 columns indicate baseline OCR vs the OCR after adding the named toxins. Mutant clones are sorted so that normal basal activity is listed first with more disrupted activity listed further down, row borders are used as a visual aid only.

Supplementary Note 1

Each experiment used on the fly training data and machine learning. Therefore, each figure has slightly different features and parameters used. Below, the details on the platform, algorithm, features, and input samples are given for each figure.

Figure 2bcd. MFN2 mutants. Platform: Azure ML Studio, Algorithm: Two-Class Boosted Decision Tree (single param, max l eaves/tree 20, min samples to form leaf 10, learning rate 0.2, #trees 100).
Features included: (8 Mitotracker Features) Mito Total Area, Nuclei Low Grey Level Run Emphasis, Mito Intensity, Cells Entropy, Nuclei Intensity CV, Cells Skewness, Cells Total Intensity (Cyto), Cells Intensity Spreading.
Dataset: Training points = 6680, Testing points = 3360, Inferred Mix points = 3638.

Figure 2ef. 289 Models. Platform: Azure ML Studio. Algorithm: Two-Class Decision Jungle (Resample by Bagging, Trainer mode from parameter range, # of decision DAGs 8/9, maximum depth 32, max width 128, # optimization steps 2048). Variable feature set with 2, 3, 5, 8, 10, 12 or 20 features, randomly subsampled from this list: (Mitotracker features) Cells Energy, Cells Entropy, Cells Grey Level Non uniformity, Cells High Grey Level Run Emphasis, Cells Intensity (Cell), Cells Intensity (Cyto), Cells Intensity CV (Cell), Cells Intensity CV (Cyto), Cells Intensity SD (Cell), Cells Intensity SD (Cyto), Cells Intensity Spreading, Cells Kurtosis, Cells Light Flux, Cells Low Grey Level Run Emphasis, Cells Max Intensity, Cells Nuc/Cyto Intensity, Cells Run Length Non uniformity, Cells Skewness, Cells Total Intensity (Cell), Cells Total Intensity (Cyto), Mito Area, Mito Chord Ratio, Mito Compactness, Mito Distance to Nuc, Mito Elongation, Mito Form Factor, Mito Gyration Radius, Mito Intensity, Mito Intensity CV, Mito Intensity Spreading, Mito Neighbor Count, Mito Org per Cell, Mito Organelle/Cyto Intensity, Mito Spacing, Mito Total Area, Mito Total Intensity, Nuclei Energy, Nuclei Entropy, Nuclei Grey Level Non uniformity, Nuclei High Grey Level Run Emphasis, Nuclei Intensity, Nuclei Intensity CV, Nuclei Intensity SD, Nuclei Kurtosis, Nuclei Light Flux, Nuclei Low Grey Level Run Emphasis, Nuclei Max Intensity, Nuclei Run Length Non uniformity, Nuclei Skewness, Nuclei Total Intensity. (Hoechst features) Cells Intensity CV (Cyto), Nuclei Skewness, Nuclei Displacement, Nuclei Grey Level Non uniformity. Training points = 6680, Testing points = 3360, Inferred Mix points = 3638.

Figure 3. Same as the above, but they have different # of features and only include the mitotracker features listed above (details in the results text and figure itself).

Figure 4bcd. MFN2 R280H. 433 models total. Platform: Azure ML Studio. Two-Class Decision Jungle (Resample by Bagging, Trainer mode from parameter range, # of decision DAGs 8/9, maximum depth 32, max width 128, # optimization steps 2048). Variable feature set with 1, 2, 3, 4, 5, 6, 8, 12, 18, 21 features, randomly subsampled from this list:
(Mitotracker features) Cells Energy, Cells Entropy, Cells Grey Level Non uniformity, Cells High Grey Level Run Emphasis, Cells Intensity (Cell), Cells Intensity (Cyto), Cells Intensity CV (Cell), Cells Intensity CV (Cyto), Cells Intensity SD (Cell), Cells Intensity SD (Cyto), Cells Intensity Spreading, Cells Kurtosis, Cells Light Flux, Cells Low Grey Level Run Emphasis, Cells Max

Intensity, Cells Nuc/Cyto Intensity, Cells Run Length Non uniformity, Cells Skewness, Cells Total Intensity (Cell), Cells Total Intensity (Cyto), Mito Area, Mito Chord Ratio, Mito Compactness, Mito Distance to Nuc, Mito Elongation, Mito Form Factor, Mito Gyration Radius, Mito Intensity, Mito Intensity CV, Mito Intensity Spreading, Mito Neighbor Count, Mito Org per Cell, Mito Organelle/Cyto Intensity, Mito Spacing, Mito Total Area, Mito Total Intensity, Nuclei Energy, Nuclei Entropy, Nuclei Grey Level Non uniformity, Nuclei High Grey Level Run Emphasis, Nuclei Intensity, Nuclei Intensity CV, Nuclei Intensity SD, Nuclei Kurtosis, Nuclei Light Flux, Nuclei Low Grey Level Run Emphasis, Nuclei Max Intensity, Nuclei Run Length Non uniformity, Nuclei Skewness, Nuclei Total Intensity. (TMRM features) Cells Intensity (Cyto), Cells Intensity CV (Cell), Cells Intensity CV (Cyto), Cells Max Intensity, Mito Intensity, Mito Intensity CV, Mito Total Intensity, Nuclei Energy, Nuclei Intensity CV, Nuclei Total Intensity. (Hoechst) Nuclei Intensity, Nuclei Area.
Two Class Logistic Regression was also used for models in this set with 10 features, sampled from the same feature set listed above. Hyperparameters (Optimization tolerance 0.0001/ 0.0000001, L1 & L2 regularization weight 1, L-BFGS Memory Size 20).
Dataset: Training points = 4472, Testing points = 2946, Inferred Mix points = 3029.

Figure 4f. MFN2 P251A. Platform: H2O.ai, which uses a Gradient Boosted Machine. It was fed the same large list of features as above, but returned 2 Mitotracker features: Cells Energy, Nuclei Intensity CV, and the TMRM feature Nuclei Energy as the most important. Training points = 2043, Testing points = 967, Inferred Mix points = 1029.

Figure 5ab. MFN2 preliminary Anomaly detection. Platform: Azure ML Studio. One Class Support Vector Machine (η = 0.1, ε = 0.001).
152 models (80 had 24 features, 56 had 32 features, 8 had 8, 8 had 16 features) randomly sampled from the list below:
(Mitotracker features) Nuclei intensity, nuclei total intensity, nuclei intensity cv, nuclei light flux, nuclei intensity sd, nuclei max intensity, nuclei skewness, nuclei kurtosis, nuclei energy, nuclei entropy, nuclei grey level non uniformity, nuclei high grey level run emphasis, nuclei low grey level run emphasis, nuclei run length non uniformity, mitonetworks intensity, mitonetworks total intensity, mitonetworks intensity cv, mitonetworks organelle/cyto intensity, mitonetworks organelle/bckg intensity, mitonetworks intensity/global bckg, mitonetworks intensity spreading, mitonetworks intensity-bckg, mitonetworks total intensity-bckg.
(Cell mask features) Cells area, cells nuc/cell area, cells form factor, cells elongation, cells compactness, cells chord ratio, cells gyration radius, cells perimeter.
(Hoechst features) Nuclei intensity, nuclei total intensity, nuclei intensity cv, nuclei light flux, nuclei intensity sd, nuclei max intensity, nuclei skewness, nuclei kurtosis, nuclei energy, nuclei entropy, nuclei grey level non uniformity, nuclei high grey level run emphasis, nuclei low grey level run emphasis, nuclei run length non uniformity.
Training points = 17946, Testing points = 64847, Inferred Points = 76573

Figure 5c. MFN2 mutagenesis screening. Platform: Scikit Learn. A dataset of 16,967 cells with known genotype and 57 normalized feature vectors* compiled over five batches were used to 479 binary classifiers. Prior to fitting, principal component analysis projected the 57 features into

11 principal components with 0.892 explained variance. The PCA embeddings were fed to a multi-dataset integration algorithm known as harmony. Harmony receives the low-dimensional embedding of the original feature vectors and learns a cell-specific linear correction function that iteratively adjusts the PCA embeddings for batch specific effects. The resulting dataset was normalized using a min-max scaler and then balanced using the synthetic minority oversampling technique. A supervised classification algorithm (either logistic regression, support vector machine, ridge regularization classifier, multinomial naïve bayes, or random forest classifier) was randomly selected, fit to a training set (80% random split), and hyperparameter tuned using a randomized search cross validation. Each model inferred a prediction probability on 59,607 cells of unknown genotype. Models were evaluated on AUC, Matthew's Correlation Coefficient, and the ability to distinguish between spiked-in positive and negative controls in the set of cells with unknown genotype. Based on these criteria, a subset of models was selected, and their prediction probabilities were averaged for each cell. This average prediction probability was used generate p-values by implementing a Benjamini-Hochberg corrected one sample t-test. The prediction probabilities generated by each model are shown in the heatmap, and the p-values computed from the average of the approved models' prediction probabilities is shown above the heatmap.

Feature list: Nuclei intensity, nuclei total intensity, nuclei intensity cv, nuclei intensity sd, nuclei max intensity, nuclei skewness, nuclei kurtosis, nuclei energy, nuclei entropy, nuclei grey level non uniformity, nuclei high grey level run emphasis, nuclei low grey level run emphasis, nuclei run length non uniformity, nuclei intensity, nuclei total intensity, nuclei intensity cv, nuclei intensity sd, nuclei max intensity, nuclei skewness, nuclei kurtosis, nuclei energy, nuclei entropy, nuclei grey level non uniformity, nuclei high grey level run emphasis, nuclei low grey level run emphasis, nuclei run length non uniformity, cells intensity (cell), cells intensity (cyto), cells intensity sd (cell), cells intensity sd (cyto), cells max intensity, cells skewness, cells kurtosis, cells energy, cells entropy, cells grey level non uniformity, cells high grey level run emphasis, cells low grey level run emphasis, cells run length non uniformity, cells intensity (cell), cells intensity (cyto), cells intensity sd (cell), cells intensity sd (cyto), cells max intensity, cells skewness, cells kurtosis, cells energy, cells entropy, cells grey level non uniformity, cells high grey level run emphasis, cells low grey level run emphasis, cells run length non uniformity, mitonetworks org per cell, mitonetworks total area, nuclei area, nuclei intensity, nuclei gyration radius.
