# Supplementary material for: Pooled image-base screening of mitochondria with microraft isolation distinguishes pathogenic mitofusin 2 mutations
Source: Commun Biol. 2022 Oct 25;5:1128. doi: 10.1038/s42003-022-04089-y (PMC9596453; doi:10.1038/s42003-022-04089-y)
Supplement: Supplementary file 2 — Description of Additional Supplementary Files [file 42003_2022_4089_MOESM2_ESM.pdf]

Description of Additional Supplementary Files

File name: Supplementary Data 1
Description: MFN2 gRNA Library

File name: Supplementary Data 2
Description: Figure Source Data for all except 5c, 5d

File name: Supplementary Data 3
Description: Fig 5c Source Data

File name: Supplementary Data 4
Description: Fig 5d Source Data
